# Cross-species identification of PIP5K1-, splicing- and ubiquitin-related pathways as potential targets for *RB1*-deficient cells

**DOI:** 10.1371/journal.pgen.1009354

**Published:** 2021-02-16

**Authors:** Andrey A. Parkhitko, Arashdeep Singh, Sharon Hsieh, Yanhui Hu, Richard Binari, Christopher J. Lord, Sridhar Hannenhalli, Colm J. Ryan, Norbert Perrimon

**Affiliations:** 1 Department of Genetics, Blavatnik Institute, Harvard Medical School, Boston, Massachusetts, United States of America; 2 Aging Institute of UPMC and the University of Pittsburgh, Pittsburgh, Pennsylvania, United States of America; 3 Cancer Data Science Laboratory, National Cancer Institute, National Institutes of Health, Bethesda, Maryland, United States of America; 4 Department of Biology, Boston University, Boston, Massachusetts, United States of America; 5 Howard Hughes Medical Institute, Boston, Massachusetts, United States of America; 6 CRUK Gene Function Laboratory, The Breast Cancer Now Toby Robins Research Centre, The Institute of Cancer Research, London, United Kingdom; 7 Systems Biology Ireland, University College Dublin, Dublin, Ireland; 8 School of Computer Science, University College Dublin, Dublin, Ireland; University of Illinois at Chicago, UNITED STATES

## Abstract

The *RB1* tumor suppressor is recurrently mutated in a variety of cancers including retinoblastomas, small cell lung cancers, triple-negative breast cancers, prostate cancers, and osteosarcomas. Finding new synthetic lethal (SL) interactions with *RB1* could lead to new approaches to treating cancers with inactivated *RB1*. We identified 95 SL partners of *RB1* based on a *Drosophila* screen for genetic modifiers of the eye phenotype caused by defects in the *RB1* ortholog, *Rbf1*. We validated 38 mammalian orthologs of *Rbf1* modifiers as RB1 SL partners in human cancer cell lines with defective *RB1* alleles. We further show that for many of the *RB1* SL genes validated in human cancer cell lines, low activity of the SL gene in human tumors, when concurrent with low levels of *RB1* was associated with improved patient survival. We investigated higher order combinatorial gene interactions by creating a novel *Drosophila* cancer model with co-occurring *Rbf1*, *Pten* and *Ras* mutations, and found that targeting RB1 SL genes in this background suppressed the dramatic tumor growth and rescued fly survival whilst having minimal effects on wild-type cells. Finally, we found that drugs targeting the identified RB1 interacting genes/pathways, such as UNC3230, PYR-41, TAK-243, isoginkgetin, madrasin, and celastrol also elicit SL in human cancer cell lines. In summary, we identified several high confidence, evolutionarily conserved, novel targets for *RB1-*deficient cells that may be further adapted for the treatment of human cancer.

## Author summary

*RB1* is a tumor suppressor gene that is inactivated in a significant proportion of all cancer cases. A therapeutic approach that specifically targets defects associated with this tumor suppressor is currently not available. A synthetic lethal (SL) interaction occurs between two genes when the inactivation of either gene alone is viable but the inactivation of both genes simultaneously results in the loss of viability. Screening for SL interactions in model organisms can identify candidate genes/pathways that can be targeted to specifically kill tumor cells with a specific mutation. Here we used a cross-species approach to identify evolutionarily conserved SL targets for *RB1*-deficient cells. We demonstrated that drugs targeting the identified targets, such as UNC3230, PYR-41, TAK-243, isoginkgetin, madrasin, and celastrol, can selectively kill human *RB1*-deficient cancer cells. Because cancer cells are characterized by the accumulation of mutations in multiple oncogenic pathways, we further confirmed that these interactions are preserved in the presence of additional strong oncogenic alterations. Moreover, low activity of the identified SL genes in human tumors, when combined with low levels of RB1, was associated with improved patient survival. Altogether, we identified several novel targets of *RB1-*deficient cells that may be further adapted for the treatment of human tumors.

## Introduction

*RB1* is a tumor suppressor gene that is frequently mutated in various tumors, including retinoblastomas, small cell lung cancers, triple-negative breast cancers, prostate cancers, and osteosarcomas. In addition, *RB1* is one of the most prevalent tumor suppressor genes driving metastasis [1]. The gene product, pRb, is also functionally inactivated in tumors with loss of cyclin-dependent kinase (Cdk) inhibitors or overexpression of Cyclin D1 or Cdk4 [2]. pRb and its family members, p107 and p130, function as inhibitors of cell cycle progression, primarily via regulation of the E2F transcription factors. pRB also interacts with SKP2 to regulate p27 levels and can bind to chromatin regulators to modulate their function. In addition, a small fraction of pRB is located in mitochondria, where it suppresses apoptosis [3–5]. Despite extensive studies of pRb family proteins, a therapeutic approach that specifically targets defects associated with this tumor suppressor is currently not available, underscoring the need for alternative strategies that can identify new targets for cancers with inactivated *RB1*.

Synthetic lethality (SL) presents a viable alternative to target RB1-mutated tumors. A pair of genes can be defined as having a SL interaction when perturbation of either gene alone is not lethal but simultaneous perturbation of both genes is [6,7]. Extending the concept of SL, Magen et al. showed that for a large number of gene pairs, their specific joint expression state (e.g., low-low, low-high, high-high, etc.) is associated with cancer patient survival [8]. There have been multiple attempts to identify genes that are SL partners of *RB1*. For example, a SL genetic screen in *Drosophila* for recessive mutations that result in the selective loss of cells lacking *dRB1* (*rbf1*) identified *CG3511*, which encodes peptidylprolyl isomerase and is a fly ortholog of human PPWD1/DIAA0073 [9]. Another genetic screen in *Drosophila* for mutations that modify the effects of *rbf1* inactivation identified *gig*, the fly ortholog of *TSC2* [10,11]. Inactivation of *Tsc2* specifically killed *Rbf1*-deficient cells in the *Drosophila* eye; induced cell death in *RB1*-mutant DU145 prostate cancer cells, Saos-2 osteosarcoma cells, and MDA-MB-468 breast cancer cells but not in their *RB1*-wild type counterparts; and inhibited tumor growth in nude mice [10,11]. In another study, inactivation of the RB1 target SKP2 prevented spontaneous tumorigenesis in *Rb1* heterozygous mice. *RB1*-deficient human retinoblastoma cells underwent apoptosis after *Skp2* knockdown [12] and small molecule inhibitors of SKP2 were SL in *RB1* defective triple negative breast tumor cells [13]. In addition, Nittner et al. showed that depletion of *Dicer1* prevented retinoblastoma formation in mice by SL with combined inactivation of *p53* and *Rb* [14]. Another SL interaction was identified between *RB1* and *MED4*. MED4 is a subunit of the mediator complex that couples specific transcription factors with RNA polymerase II. Retinoblastoma *RB1*-deficient cells were not able to survive in the absence of *MED4 in vitro* and in a xenograft mouse model in *vivo* [15]. Bioinformatics analysis integrating molecular profiling data with data from multiple genetic perturbation screens also identified *SKP2* as a *RB1*- SL target, as well as nuclear pore complex components (NUP88 and NUP214) and the bromodomain containing transcription factor TAF1 [13]. Further, a pharmacogenomic screen identified LY3295668, a highly specific Aurora A inhibitor that specifically kills *RB1*-deficient cancer cells [16]. Similarly, a SL CRISPR/Cas9 screen in an *RB1−/−* SCLC cell line with inducible expression of RB1 showed that Aurora B kinase or its kinase inhibitor, AZD2811, were efficacious in multiple preclinical SCLC models [17]. In addition, *RB1* mutations have been shown to sensitize cancer cells to the mitotic inhibitors Taxol and STLC [18]. Several genome-scale shRNA and CRISPR screens have also been performed in large panels of human cancer cell lines (irrespective of their mutational status). These cell lines can be bioinformatically distinguished based on their *RB1* status to identify genes that more effectively target *RB1-*deficient cells [19–22]. Despite these studies, the numerous SL partners of RB1 have unfortunately not yet led to a targeted approach to treating RB1 defective cancers, underscoring the need for additional candidates.

To identify novel high-confidence therapeutic targets in the RB1 pathway, we systematically combined several of the aforementioned approaches. First, we performed a SL genetic screen in the *Drosophila* eye for modifiers of an *Rbf1*-deficient eye phenotype. Next, we further filtered this list to only retain orthologs of these genes that more efficiently suppress proliferation of human *RB1*-deficient cancer cell lines, as identified based on publicly available large-scale shRNA and CRISPR screen data. To prioritize genes of potential clinical relevance, we applied the recently developed SPAGE-finder algorithm [8] to identify genes whose low expression, when combined with low *RB1* expression, is associated with improved patient survival. To further investigate higher-order combinatorial gene interactions involving Rbf1, we created a new *Drosophila* tumor model in which *Rbf1* deficiency is combined with loss of *Pten* and overexpression of activated *Ras1A* in adult intestine stem cells (ISCs), which results in dramatic fly gut overgrowth and reduced lifespan. We next confirmed that hits uncovered in the *Drosophila* eye screen and human cancer cell lines efficiently suppress growth of these tumor cells, with moderate to no effect on wild-type cells. Finally, we demonstrate that chemical inhibitors of the pathways enriched among RB1 SL partners that we identified, such as UNC3230, PYR-41, TAK-243, isoginkgetin, madrasin, and celastrol, more efficiently suppressed proliferation of *RB1*-deficient cancer cell lines compared to cells with intact *RB1*. In conclusion, via systematic multi-pronged approach, we identified novel SL interactors with *RB1* and propose that potential genes/targets that interacted with *RB1* orthologs in *Drosophila* and interact in human cancer cell lines and patient tumor samples, and drugs that inactivate these targets are more likely to have an effect in patients with *RB1*-mutant cancers.

## Results

### *Drosophila* eye screen for synthetic lethality with *Rb1* deficiency

**Fig 1:**
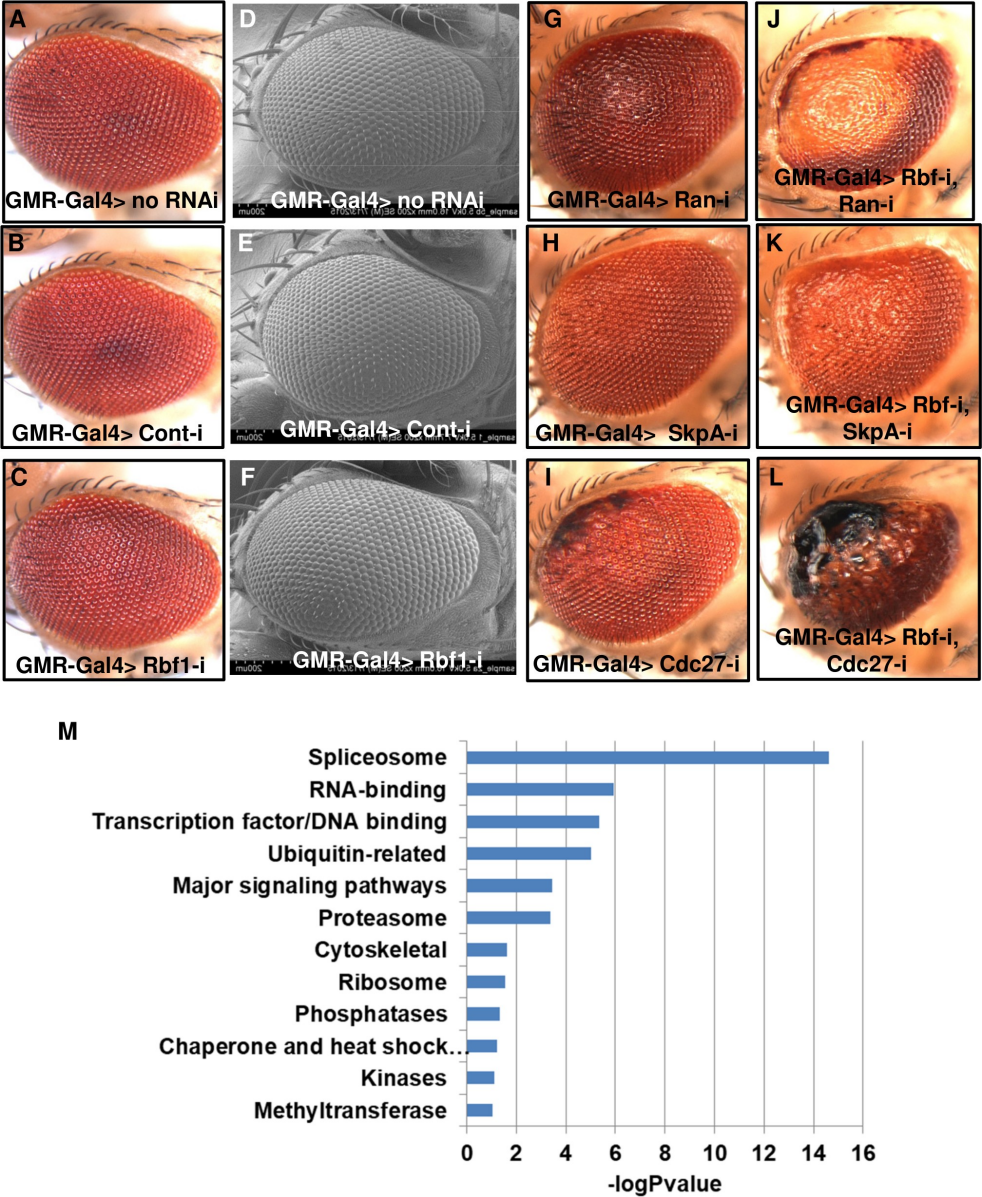
*Drosophila* synthetic lethality eye screen in *Rb1*-deficient background. *Drosophila* eye pictures of the following genotypes: *GMR-Gal4> no RNAi* **(A)** *GMR-Gal4> Cont-i* **(B)** *GMR-Gal4> Rbf1-i* **(C)**. Scanning electron microscope images of a *Drosophila* eye of the following phenotypes: *GMR-Gal4> no RNAi* **(D)** *GMR-Gal4> Cont-i* **(E)** GMR-Gal4*> Rbf1-i* **(F)**. *Drosophila* eye picture of the following phenotypes: *GMR-Gal4> Ran* RNAi **(G)**, *GMR-Gal4> SkpA* RNAi **(H)**, *GMR-Gal4*> *Cdc27* RNAi **(I)**, *GMR-Gal4*> *Rbf1-i*, *Ran* RNAi **(J)**, *GMR-Gal4*> *Rbf1*-i, *SkpA* RNAi **(K)**, *GMR-Gal4*> *Rbf1*-i, *Cdc27* RNAi **(L)**. GO enrichment analysis of *Drosophila* eye hits **(M)**.

The *Drosophila* eye has previously been used to screen for mutations that result in the loss of d*Rb-*deficient but not wild-type cells [9,11]. We created fly lines with either control RNAi (*GMR-Gal4>Cont-i*) or previously validated RNAi reagents against *Rbf1* [23] (*GMR-Gal4>Rbf1-i*) driven by the eye-specific driver Glass Multimer Reporter-Gal4 *(GMR-Gal4*) [24]. We further crossed control or *Rbf1* RNAi lines with 1300 RNAi lines targeting genes identified as orthologs of genes from various cancer-related sets (**S1 Table**). As GMR-Gal4 drives expression mostly in post-mitotic cells, we did not expect to identify large overgrowth phenotypes; although previous studies have reported that GMR- or GMR-Gal4-driven expression of cancer-relevant proteins can promote proliferation both autonomously [25,26] as well as non-autonomously [27]. Instead, we expected to identify RNAi lines which in combination with *GMR-Gal4>Rbf1-i* would cause neuronal death, as Khurana et al. have shown that reactivation of the cell cycle leads to neuronal death [28]. Thus, we expected that *Rbf1* loss of function together with downregulation of genes from the selected library that reactivate the cell cycle would lead to cell death in the eye (and lead to glossy, rough, smoothened, or darkened phenotypes). Also, since pRb is involved in numerous cancer hallmark pathways [29], besides G1/S transition/cell cycle control, such as metabolism, mitochondrial function, apoptosis, epigenetic state, and differentiation [3], our sensitized Rbf1 eye screen aimed at identifying a broad spectrum of Rbf1 genetic interactors using either overproliferation phenotypes, cell death phenotypes associated with activation of the cell cycle, or a diverse set of cell cycle-independent phenotypes. *GMR-Gal4* flies crossed with no RNAi, control RNAi or *Rbf1* RNAi do not have visible eye phenotypes or retinal degeneration (**Fig 1A–1F**). In the screen, we identified several genes that cause very strong phenotypes in both control and *Rbf1*-deficient background (i.e. genes that exhibit general toxicity). In addition, we identified 95 genes that do not have a phenotype (or very moderate phenotypes) when combined with a control RNAi but have a phenotype (or have a stronger phenotype) when combined with *Rbf1* RNAi. The specific eye phenotypes we observed were different for different interactors and included glossy, rough, smoothened, or darkened eyes (**Figs 1G–1L and S1–S8**). Following the screen, we performed a Gene Ontology enrichment analysis (**Fig 1M**) in an effort to understand which biological processes are most relevant to genetic interaction with *Rbf1* deficiency. The enrichment analysis revealed members of regulators of cytoskeleton or proteasome GO groups, the spliceosomal complex and ubiquitin-related pathways among the top groups interact with *Rbf1* deficiency. Altogether, in the screen we identified 95 genes whose disruption might selectively target *Rbf1*-deficient cells.

### Identification of a high-confidence gene subset based on large-scale screens in human cancer cell lines

**Fig 2:**
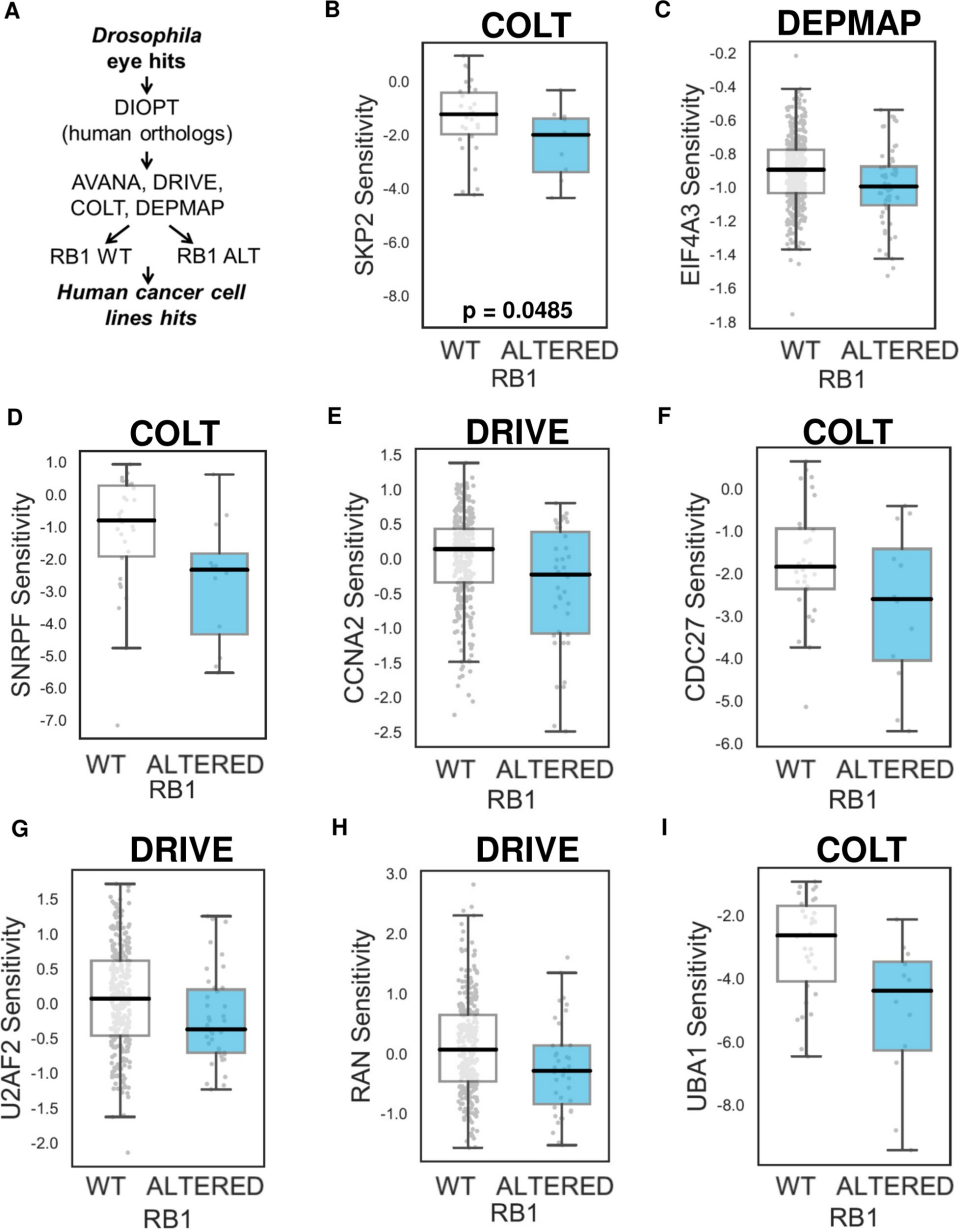
Testing human gene orthologs of the *Drosophila* hits in available large-scale shRNA and CRISPR human cancer cell line screens. Data analysis workflow **(A)**. Scatter plots illustrating Z scores in DRIVE, AVANA, COLT, DEPMAP data sets in *RB1* wild-type (WT) and mutant (ALT) human cancer cell lines for candidate RB1 SL partners: *SKP2* **(B)**, *EIF4A3* **(C)**, *SNRPF* **(D)**, *CCNA2* **(E)**, *CDC27* **(F)**, *U2AF2* **(G)**, *RAN* **(H)**, and *UBA1* **(I)**.

Several large-scale shRNA or CRISPR-Cas9 mutagenesis screens have been performed in large panels of tumor cell lines [19–22]. These large-scale screens allow genetic dependencies (e.g. SL effects) associated with specific tumor suppressor gene or oncogene alterations to be identified in cancer cell lines with heterogeneous molecular compositions. We analyzed data from the following projects: COLT [22], DRIVE [19], AVANA [20], and DEPMAP [21]. The COLT project performed a whole-genome shRNA screen on 77 breast cancer cell lines [22]. In the DRIVE project, a large-scale viability-based shRNA screen targeting 7,837 genes was performed in 398 cancer cell lines. Bioinformatics analysis revealed that cell lines with amplification of E2F3 transcription factor and/or with loss of expression of RB1 were found to be dependent on downregulation of E2F3, SKP2, CKS1B, or CDK2 [19]. In the AVANA project, genome wide CRISPR-Cas9 screens were performed across 517 cancer cell lines [20]. Finally, in the DEMETER project, an analytical framework for the systematic identification of cancer dependencies was generated as a result of 501 genome-scale loss-of-function screens performed in diverse human cancer cell lines [21]. To identify which out of the 95 genes we identified in the *Drosophila* eye screen represent true dependencies for *RB1*-deficient cells, we interrogated the effects of the depletion of their human orthologs on the proliferation of human cancer cell lines from the COLT, DRIVE, AVANA, and DEPMAP projects based on their *RB1* status (**Fig 2A)**. We started by performing this analysis on 12 genes that have been previously shown to suppress growth of *RB1*-deficient cells in different contexts (discussed in the introduction section). As expected, downregulation of *SKP2*, a downstream target of RB1, more efficiently suppressed the proliferation of *RB1*-deficient cancer cell lines in all 4 screens (**Fig 2B**) suggesting that RB1/SKP2 is a relatively robust SL interaction, being independent of the gene perturbation approach used (shRNA or CRISPR-Cas9) or the somewhat differing cell line panels used in different projects. In addition, downregulation of *E2F3* scored in all 4 screens, *E2F1* and *NUP214* in 2 screens, *AURKB*, *NUP88*, and *TAF1* in 1 screen (**S2 Table**). We next assessed whether human orthologs of the hits identified in our *Drosophila* screen were also SL with *RB1* loss in human cancer cell lines. Due to gene duplication events, the 95 hits that we identified in *Drosophila* map to 164 human orthologs. Of these 164 human genes, 2 genes were identified as *RB1* dependencies in 3 screens (Human/*Drosophila*): EIF4A3/ eIF4AIII and SNRPF/ SmF (**Fig 2C and 2D**); 7 genes scored in 2 screens (Human/*Drosophila*): RAN/Ran, PSMA3/ Prosalpha7, UBA1/ Uba1, CCNA2/ CycA, POLR2D/Ada2a,Rpb4, U2AF2/ U2af50, and CDC27/ Cdc27 (**Fig 2E–2I**); and 29 genes scored in 1 screen (**S2 Table**). Thus a total of 38 putative human *RB1* dependencies were identified among the orthologs of our *Drosophila* screen hits. To determine if this was more than would be expected by chance, we repeatedly sampled 164 human genes with orthologs in our *Drosophila* library and assessed their overlap with hits identified in cancer cell lines. Across 1,000 random samples we found an average overlap of 19 and a maximum overlap of 31, suggesting that the overlap between our *Drosophila* screen hits and those observed in cancer cell lines was significantly more than random expectation (nominal p-value < 0.001).

Among genes that scored in only 1 screen, especially interesting ones were PIP5K1C/Sktl and RNF2/ Sce that scored in the AVANA screen. AVANA is a CRISPR/Cas9 screen, suggesting that mutagenesis or complete loss of function of these genes might elicit SL, whilst partial inhibition, for example, by shRNA/siRNA might not. Interestingly, when Oser et al. re-expressed RB1 in *RB1*-deficient NCI-H82 SCLC cancer cell line and performed a CRISPR/Cas9 pRB SL screen [17], they also identified Ran, SNRPF, multiple POLR members and U2AF1. Both U2AF1 (from Oser’s screen) and U2AF2 (from our screen) form a heterodimer and have a similar function further confirming the validity of the hits that we selected from *Drosophila* and human screens. It should also be noted that it is unlikely that the same hits would produce SL interactions across all *RB1*-deficient tumor types. We instead expect that different tumor types will be more sensitive for specific hits. In summary, our cross-species data integration based on data from the *Drosophila* eye screen and human cancer cell line screens identified 38 genes as potential therapeutic targets for *RB1*-deficient cancer cells.

### Identifying synthetic lethal partners of *RB1* in human cancer patients

**Fig 3:**
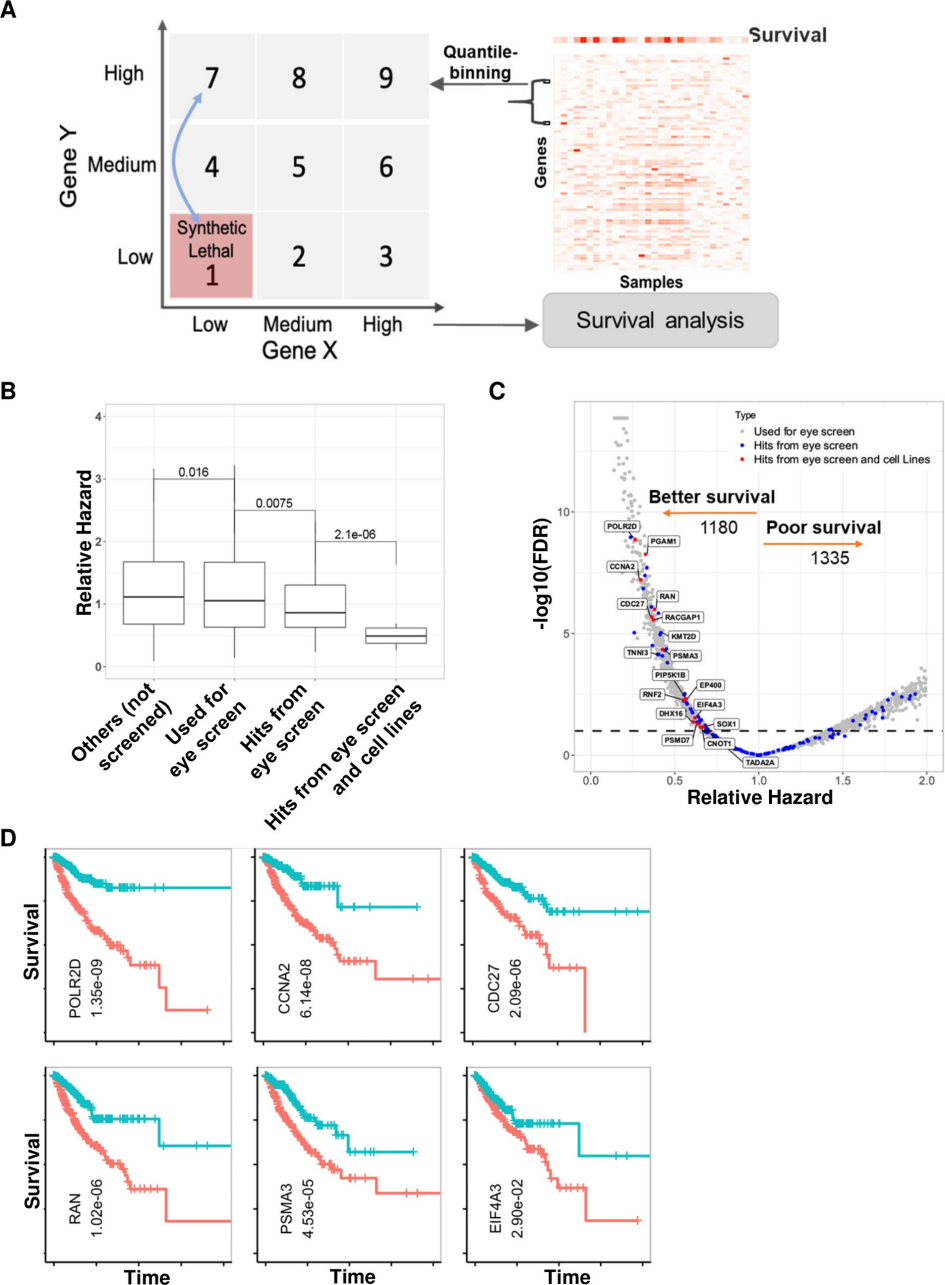
Identifying synthetic lethal partners of *RB1* in human cancer patients. (**A**) We analyzed the overall survival of the TCGA cancer patients by comparing the patients with low activity of both genes (bin 1) against the low activity of *RB1* and high activity of candidate partner gene (bin7) in pooled lung, breast, and prostate cancers patients. (B) Box plot comparing the relative hazard computed using log-rank test for different gene sets. Gene sets from left to right represent (i) 17155 genes not used in the *Drosophila* screen, (ii) the human orthologs of 1300 genes used in the *Drosophila* screen, (iii) human orthologs of 95 genes identified as RB1 SL partners in the *Drosophila* screen, and (iv) 18 genes identified as RB1 SL partners in cancer cell lines. **(C)** Volcano plot showing relative hazard and–log10(p-value) for human orthologs of the genes that we screened in *Drosophila*, with human orthologs of hits from the *Drosophila* eye screen and cell line hits shown with blue and red colors, respectively. The p-value was adjusted for multiple comparisons using Benjamini-Hochberg’s FDR method. Dotted horizontal line indicates the FDR of 10%, arrows indicate direction of survival and the numbers below arrows indicate the number of genes having better or worst survival in conjunction with low RB1. (**D**) Kaplan Meier’s survival curves along with corrected p-values for six genes which showed SL with *RB1* in cancer patients and in at least two human cancer cell lines screens. Blue line–samples in bin 1, Red line–samples in bin 7.

To test the clinical relevance of *Drosophila* SL pairs of *RB1* in human cancer patients, we used SPAGEfinder [8], which identifies pairwise gene expression states that are significantly associated with survival of cancer patients. We used the gene expression and clinical data for lung, breast and prostate cancers from TCGA. In SPAGEfinder, the expression of each gene across tumor samples is divided into three activity states: low, medium, and high, resulting in a total of 9 possible joint activity states (interaction bins) between two genes (**Fig 3A**). Since we were only interested in SL interactions with *RB1*, we compared the overall survival of the patients who had simultaneous downregulation of *RB1* and a candidate gene (Bin1 in Fig 3A) against the patients where only *RB1* is downregulated but its partner gene is not (Bin7 in Fig 3A). To quantify the difference in survival, we calculated the relative hazard of patients in Bin1 against those in Bin7 using log-rank test. Interestingly, human orthologs of genes scored as hits in the *Drosophila* eye screen had significantly lesser hazard as compared to human orthologs of genes that we tested in the *Drosophila* screen or other genes in the rest of the genome (**Fig 3B**). Moreover, 18 genes (*EIF4A3*, *RAN*, *PSMA3*, *CCNA2*, *POLR2D*, *CDC27*, *PGAM1*, *RACGAP1*, *PIP5K1B*, *DHX16*, *EP400*, *RNF2*, *PSMD7*, *CNOT1*, *SOX1*, *TADA2A*, *TNNI3*, *KMT2D*) that scored as hits in cancer cell screens had least hazard than any other group of genes (**Fig 3B**) and better survival (Bin1 vs Bin7) at FDR threshold of 10% (**Fig 3C**). In our analysis, 6 out of 18 genes (*EIF4A3*, *RAN*, *PSMA3*, *CCNA2*, *POLR2D*, *CDC27*) were scored as SL hits against *RB1* in at least two human cancer cell lines screens. In the TCGA data, we found that patients with downregulation of *RB1* along with downregulation of any of these six genes had better survival compared to patients with downregulation of *RB1* alone (**Fig 3D**); the same trend was observed for the 12 genes identified in any one of human cancer cell lines screens (**S9A Fig**). Interestingly, we found several cases when a *Drosophila* gene has multiple human orthologs and one of this orthologs scored as a hit in the analysis of human cancer cell lines and another ortholog scored as a hit in human cancer patients. As an example, the *Drosophila* gene *Taf1* that scored as a hit in the eye screen has two orthologs in humans: *TAF1* and *TAF1L*. *TAF1* scored as a SL *RB1* partner in human cancer cell lines (COLT, as previously described [13]) and *TAF1L* in human cancer patients. Other genes with the similar pattern include human orthologs of *Drosophila Hsc70-1*—*HSPA1B* (scored in human cancer cell lines) and *HSPA6* (scored in human cancer patients); *Pglym78*—*PGAM1*, *PGAM2* (scored in human cancer cell lines) and *PGAM1*, *PGAM4* (scored in human cancer patients); *CG31871* –*LIPM* (scored in human cancer cell lines) and *LIPA* (scored in human cancer patients); sktl—*PIP5K1B*, *PIP5K1C* (scored in human cancer cell lines) and *PIP5K1A*, *PIP5K1B* (scored in human cancer patients) (**S2 Table**). One reason could be that downregulation of one of mammalian orthologs might be compensated by others. Among 12 genes reported in the literature as SL with *RB1* 6 genes (*PPWD1*, *SKP2*, *AURKA*, *AURKB*, *E2F1*, and *E2F3)* scored as SL *RB1* partners in human cancer patients (**S2 Table** and **S9B Fig**). We further note that our approach used *RB1* mRNA level as a proxy for its activity, even though its activity might be attenuated post-translationally due to modifiers. The resulting inaccuracy in ascribing high RB1 activity to a sample may result in false negatives but not likely to result in false positives.

We further extended our analysis on other members of RB1 pathway, including *CDKN2A* (p16Ink4a), *CCND1*, *CDK4*, and *CDK6*, which are often mutated in different cancers. Similar to *RB1*, genes that scored as hits both in the *Drosophila* eye screen and cancer cell line screens had least hazard than any other group of genes in patients with low expression levels of *CDKN2A* (*p16Ink4a*) in tumor samples (**S10 Fig**). In summary, our results suggest that many of the genes found to be SL partners of *RB1* in the *Drosophila* screen and human cancer cell lines may also be associated with survival outcomes in human patients whose tumors have lost RB1 expression.

### Protein network analysis of synthetic lethal *RB1* partners from *Drosophila*, human cancer cell lines, and human cancer patients

**Fig 4:**
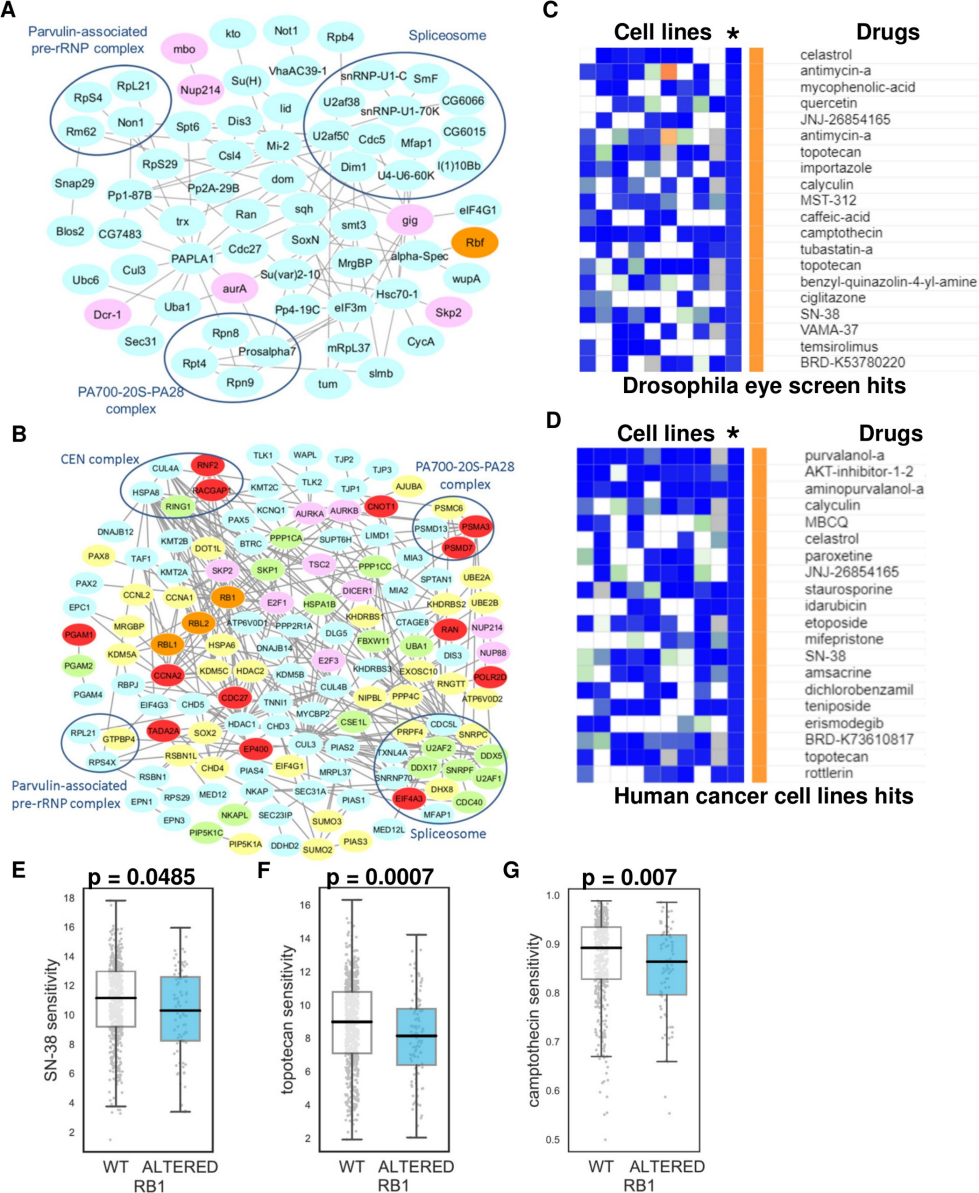
Protein network analysis of synthetic lethal RB1 partners from *Drosophila*, human cancer cell lines, and human cancer patients. **(A)** PPI network of *Drosophila* eye hits (marked in blue color), positive controls from the literature (marked in violet) and Rbf gene (marked in orange). **(B)** PPI network of the full list of human orthologs of *Drosophila* eye hits and positive controls from literature; subnetwork 1 contains proteins that also scored as SL partners in human cancer cell lines (marked in green or red); subnetwork 2 contains proteins that also scored as SL partners in human cancer patients (marked in yellow or red nodes); the common genes from subnetwork 1 and 2 are marked in red. **(C, D)** Top 20 drugs from the connectivity map analysis that scored from the analysis of the full list of human orthologs of *Drosophila* eye hits and positive controls from literature **(C);** and from the analysis of subnetwork 1 that contains proteins that also scored as SL partners in human cancer cell lines **(D)**. Columns—cMap cell lines, rows–drugs. Decreased gene expression signature is marked in blue and increased–in red. *—marks an average of gene expression signature between all tested cell lines. Scatter plots illustrating Z scores for SN-38 **(E)**, topotecan **(F)**, and camptothecin **(G)** in *RB1* wild-type and mutant cell lines retrieved from CTRP.

Previous analyses, especially of the yeast genetic interaction network, have demonstrated that the SL partners of an individual gene are often densely connected on the protein-protein interaction network [30–32]. This suggests that loss of one gene often results in an increased sensitivity to the inhibition of entire pathways or complexes. This enrichment of protein-protein interactions among SL partners has been proposed as an additional validation of identifying true positive hits from functional screens [33,34]. In order to identify whether the hits from our *Drosophila* eye screen were enriched in protein-protein interactions we integrated them with a *Drosophila* protein-protein interaction network [34]. We found high connectivity among the hits; i.e. 91 interactions among the 95 hits, excluding self-interactions (**Fig 4A**); this is significantly higher compared to connectivity among 95 randomly selected genes from all screened genes, varying from 6 to 81, with an average of 30 interactions across 1000 trials corresponding to nominal p-value < 0.001 (**S11A Fig**). We also analyzed whether our hits are connected to other known RB1 SL partners, as well as to Rbf1 or Rbf2. We found that 14 of 95 hits interact with other known RB1 SL partners (**Fig 4A)**. In comparison, the average number of interacting genes from randomly generated gene lists was 8. We next analyzed protein connectivity of human orthologs and highlighted proteins that also scored as SL partners in human cancer cell lines and in human cancer patients (**Fig 4B).** Similar to the *Drosophila* network, most of the human proteins were more interconnected (161 interactions) comparing to the 1000 list of random selected genes from target human gene list ranging from 36–201 with an average of 93 interactions.

To test the potential clinical utility of the identified SL *RB1* partners, we took two approaches: first, we searched the literature for small molecules that have been shown to target protein products of *RB1* SL partners (see below) and second, we utilized the Connectivity Map to search for drugs that cause a strong transcriptional response towards all sets of genes that we identified as potential SL partners. The Connectivity Map (CMap) contains gene expression signatures of a variety of cell types treated with several thousands of small molecule drugs [35,36]. We analyzed either the full list of all human orthologs that scored in the *Drosophila* eye screen (**Fig 4C and S3 Table**), a subset of human orthologs that scored in the *Drosophila* eye screen and human cancer cell lines (**Fig 4D and S3 Table**), a subset of human orthologs that scored in the *Drosophila* eye screen and human cancer patients (**S11B Fig and S3 Table**), and in the *Drosophila* eye screen, human cancer cell lines, and human cancer patients (**S11C Fig and S3 Table**). From each analysis, we selected the top 20 small molecule drugs that represent potential drugs for *RB1*-deficient cancers based on their inhibitory transcriptional effect on genes that function as *RB1* SL partners. We obtained drug sensitivity profiles for 29 of these compounds from three different drug screening resources: CTRP, GDSC, and PRISM [37–39] (**S4 Table**). We found that three compounds appeared to display selectivity for *RB1* mutant cell lines, the topoisomerase poisons topotecan (p = 0.00004, FDR = 0.00067), SN-38 (p = 0.006, FDR = 0.0485), and camptothecin (p = 0.0006, FDR = 0.0075) (**Fig 4E–4G** and **S4 Table**). Topotecan was found to selectively inhibit the growth of *RB1* mutant cell lines in both the GDSC resource and the CTRP resource, suggesting that it is a robust effect. This is further supported by the fact that the combination of topotecan and vincristine was effective for the treatment of advanced intraocular retinoblastoma [40]. Moreover, *RB1* loss (in combination with impaired BRCA-mutant signature and high SLFN11 expression) was highly predictive for the response of patient-derived xenografts of triple-negative breast cancer to irinotecan (SN-38 is the active metabolite of irinotecan) [41]. Also, other topoisomerase inhibitors, notably etoposide (that also scored as a hit in the CMap analysis in three different subsets), have been shown to elicit RB1 SL [42]. Interestingly, we found that two drugs (celastrol and calyculin) were retrieved from all 4 subsets and three drugs (etoposide, idarubicin, JNJ-26854165/Serdemetan, and purvalanol-a) were retrieved from 3 different subsets as potential hits based on the CMap analysis. In summary, we found high connectivity of the candidate hits and identified a potential approach to target this network as a whole using small molecule drugs from the Cmap analysis.

### Creation of a novel Rbf1/Pten/Ras mutant *Drosophila* cancer model

**Fig 5:**
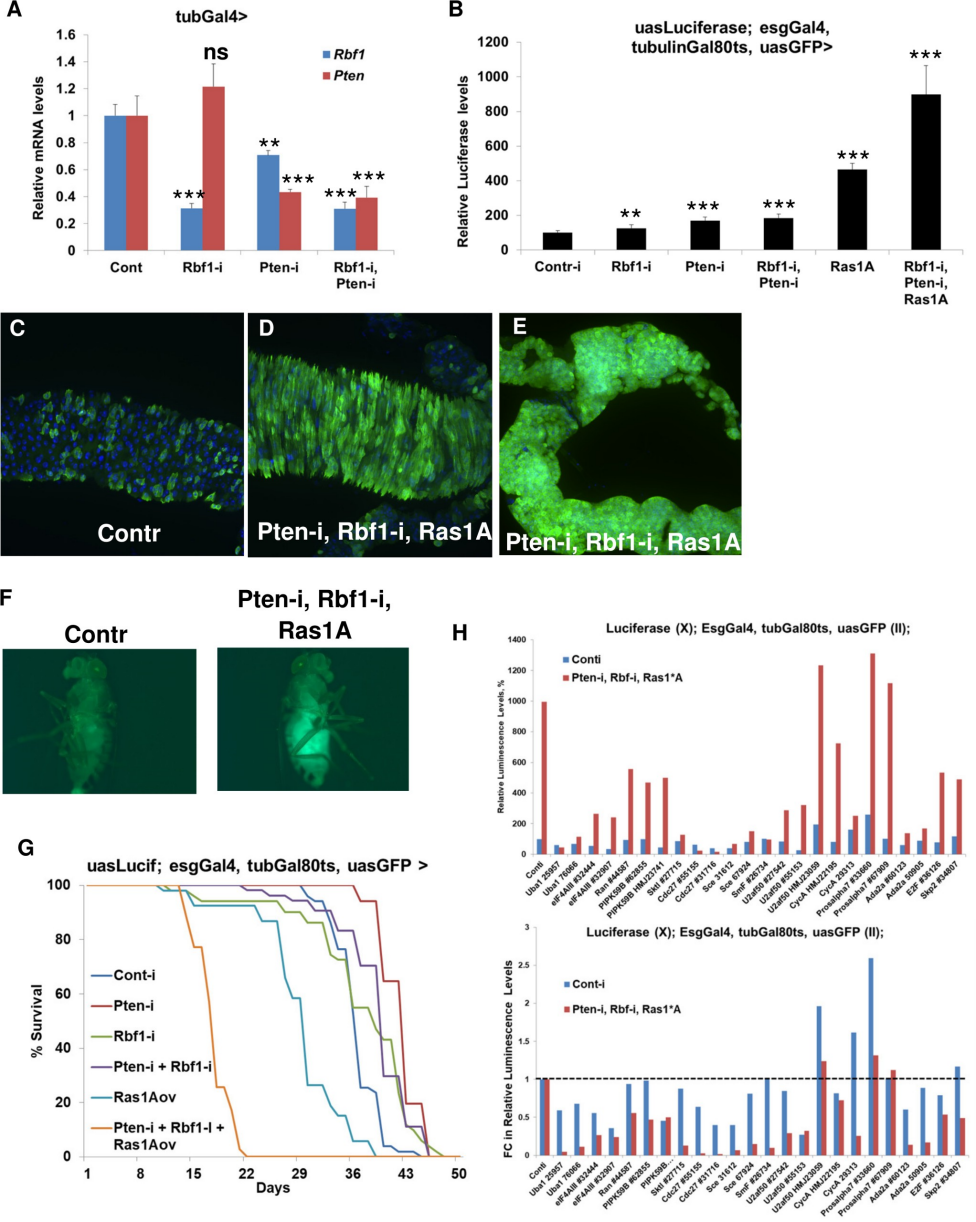
Creation of a novel Rbf1/Pten/Ras *Drosophila* cancer model. **(A)** Relative mRNA levels of *Pten* and *Rbf1* in *tubulin-Gal4* larvae expressing either control RNAi, *Rbf1* RNAi, *Pten* RNAi or double *Rbf1*, *Pten* RNAi. Means ± SD. **p<0.01, ***p<0.001 **(B)** Relative luciferase levels in *UAS-Luciferase*,*esg-Gal4*,*tubulinGal80ts*,*UAS-GFP* female flies expressing either control RNAi, *Rbf1* RNAi, *Pten* RNAi, double *Rbf1*,*Pten* RNAi, activated *UAS-Ras1A*, or combined *Rbf1*,*Pten RNAi*,*Ras1A*. Means ± SD. **p<0.01, ***p<0.001. Maximum projection of confocal images of posterior midgut **(C, D)** and Malpighian tubules **(E)** of *UAS-Luciferase*, *esg-Gal4*, *tubulinGal80ts*, *UAS-GFP* flies crossed to control RNAi **(C)** or combined *Rbf1*, *Pten* RNAi,*Ras1A* **(D, E)**. Whole fly pictures under fluorescent microscope of *UAS-Luciferase*, *esg-Gal4*, *tubulinGal80ts*, *UAS-GFP* flies crossed to either control RNAi or combined *Rbf1*, *Pten RNAi*, *Ras1A* **(F).** Lifespan analysis of *UAS-Luciferase*, *esg-Gal4*, *tubulinGal80ts*, *UAS-GFP* flies crossed to either *control RNAi*, *Rbf1 RNAi*, *Pten RNAi*, double *Rbf1*, *Pten RNAi*, *activated UAS-Ras1A*, or combined *Rbf1*, *Pten RNAi*, *Ras1A* **(G)**. Absolute (top) and relative (bottom) luciferase levels in two tester lines: *UAS-Luciferase*, *esg-Gal4*, *tubulinGal80ts*, *UAS-GFP* lines containing either control RNAi (control) or combination of *Rbf1* RNAi, *Pten* RNAi, *Ras1A* overexpression and crossed to either control RNAi or RNAi against 11 genes and *E2F* and *Skp2* as positive controls. Four biological replicates/genotype. **(H)**.

Tumor progression, metastasis, and acquired drug resistance are more often characterized by the accumulation of mutations in multiple oncogenic signaling pathways, as opposed to mutations in one gene, suggesting that they are polygenic, rather than monogenic, phenotypes [1,43,44]. We hypothesized that the accumulation of additional mutations might prevent response of *RB1*-deficient cells to targeting of the SL partners that we identified. We therefore extended the notion of SL between a pair of genes to a higher order, by assessing SL in the background of multiple co-occurring oncogenic perturbations. Similar higher-order effects have been previously studied in yeast [45] and the BRCA1/PARP inhibitor SL interaction can be rescued by compensating alterations in other genes such as *53BP1* [46]. Toward this, we created a new *Drosophila* cancer model in which downregulation of *Rbf1* is combined with downregulation of *Pten* and overexpression of Ras (*Drosophila* Ras85D is a single high-scoring ortholog of human KRAS, HRAS, and NRAS). Several reasons led us to build this specific model: (1) human orthologs of these genes are co-mutated in subsets of human cancer samples [47,48]; (2) alterations in the canonical pathways to which these genes are attributed, i.e. RB1, cell cycle; PTEN, PI3K/Akt signaling, and RAS, RTK-RAS signaling, co-occur in multiple cancer types [43]; (3) mutations in these three genes are among the most significant drivers of metastasis [1]; and (4) functional interaction, in terms of tumorigenesis, between orthologs of these genes has previously been demonstrated in mouse models. To create triple transgenic flies, we used a previously developed UAS-containing vector [49]. We cloned *Rbf1* and *Pten* RNAi sequences into a UAS vector containing a *ftz* intron in front of eGFP and created transgenic flies at the attP2 site. To compare the knockdown efficiency of single and double *Rbf1*, *Pten* RNAi lines, we used the ubiquitous *TubulinGal4* driver. The knockdown efficiency was similar between single RNAi lines and the double *Rbf1* RNAi, *Pten* RNAi line **(Fig 5A)**. We then recombined double *Rbf1* RNAi, *Pten* RNAi line with activated *UAS-Ras1A* (*Ras1A* overexpression [*Ras1A-OE*]) [50,51]. To test the functional consequences of this combined genotype, we crossed either single or combined *Rbf1* RNAi, *Pten RNAi*, *Ras1A-OE* with a previously developed *UAS-Luciferase*, *esg-Gal4*, *tubulinGal80ts*, *UAS-GFP* line [52]. Gal80ts is active at 18°C and represses Gal4, whereas at 29°C, Gal80ts is inactivated, allowing Gal4-dependent expression, in this case in *esg*-positive cells, i.e. intestinal stem cells (ISCs) and enteroblasts in the gut, as well as Malpighian tubule stem cells. When flies are switched from 18°C to 29°C, Gal4 drives expression of the UAS-transgene (either control single reagents or the triple transgene), GFP (allowing visualization of Gal4-positive cells), and Luciferase (allowing us to quantify Gal4-positive cells). Downregulation of *Rbf1* or *Pten* significantly but very moderately increased the number of GFP/Luciferase-positive cells, whereas Ras1A overexpression had a stronger effect (**Fig 5B**). The combination of *Rbf1* RNAi, *Pten* RNAi, and *Ras1A-OE* caused the strongest (~10-fold) increase in the number of GFP/Luciferase-positive cells and made the posterior part of midgut completely filled with GFP/Luciferase-positive cells (**Fig 5B–5D**). Moreover, the combination of *Rbf1* RNAi, *Pten* RNAi, *Ras1A-OE* caused dramatic overgrowth of the Malpighian tubules (**Fig 5E**). Tumor growth in *Rbf1* RNAi, *Pten* RNAi, *Ras1A-OE* was so significant that it could be observed even without gut dissection in intact flies (**Fig 5F**). In addition to tumor size, another critical characteristic in clinical oncology is overall survival (OS) of cancer patients. To test how downregulation of *Rbf1* or *Pten*, ectopic activated *Ras1A*, or their combinations affect OS, we measured fly lifespan after inducing tumor growth. Downregulation of *Pten* alone, *Rbf1* alone, and the *Pten*, *Rbf1* combination did not affect OS. However, overexpression of *Ras1A* significantly suppressed OS, and the combination of *Rbf1* RNAi, *Pten* RNAi, and *Ras1A-OE* dramatically shortened the OS of flies (**Fig 5G**). We next combined the model with RNAi knockdown of candidates. Because our goal was to test if these are still effective in the context of additional oncogenic alterations, we did not assess all possible combinations, and instead, focused on testing of a control and the *Rbf1* RNAi, *Pten* RNAi, *Ras1A*-OE tumor model. We chose to test whether 11 genes that serve as potential therapeutic targets for *RB1*-deficient cancer cells in different settings can also suppress the growth of tumors with additional alterations. We first created two tester lines: *UAS-Luciferase*, *esg-Gal4*, *tubulinGal80ts*, *UAS-GFP* lines with a control RNAi (control) or with a combination of *Rbf1* RNAi, *Pten* RNAi, and *Ras1A-OE* (tumorigenic line). We then crossed RNAi lines corresponding to the 11 target genes to the tester stocks and monitored tumor growth using a Luciferase readout **(Fig 5H)**. To compare control and tumor cells, we normalized control levels to 100% and considered *Rbf1*-deficient tumors cells to be more sensitive when downregulation of one of the 11 genes caused greater than a 25% decrease in tumor cells as compared to the control (**Fig 5H, bottom panel**). We found that 10 of 11 genes (all except *Prosalpha7*) identified in our pipeline effectively inhibited proliferation of tumor cells, as did the positive controls Skp2 and E2F. Although downregulation of several of these hits significantly affected the number of Luciferase-positive cells in control flies, the effect was much stronger in tumor flies, hinting at a possible therapeutic window that could be exploited to selectively kill tumor cells. Although we cannot exclude the possibility that *Ras1A* overexpression and/or downregulation of *Pten* may also contribute to the observed phenotype, it is possible that their manipulations lead to the functional inactivation of pRb and this may phenocopy the effect of genetic inactivation of *Rbf1*. More importantly, our goal was to test whether the SL interactions between 11 candidates and inactivation of Rbf1 is preserved in the context of multiple oncogenic alterations and in the context of strong tumor overgrowth that was confirmed by our data. In *Drosophila*, genetic conditions that strongly repress ISC proliferation are deleterious and shorten lifespan, while limiting age-dependent ISC overproliferation and intestinal tumorigenesis extends lifespan [53]. In addition, therapeutic interventions that slow down tumor growth but do not extend overall survival are not likely to be approved for the use in humans. We further tested whether selected hits (Uba1, Cdc27, Ada2a/Rpb4, or eIF4AIII) that we identified affect lifespan of control flies or flies with the combination of *Rbf1* RNAi, *Pten* RNAi, *Ras1A-OE*. We found that none of them decreased lifespan of control flies but their downregulation significantly rescued the lifespan of flies with the dramatic tumor overgrowth (S12A–S12D Fig). Interestingly, downregulation of Cdc27, Ada2a/Rpb4, or eIF4AIII increased lifespans of control flies, an effect that may be associated with suppression of age-dependent ISC overproliferation. In summary, we created a new *Drosophila* cancer model characterized by strong tumor overgrowth in the intestine and Malpighian tubules that dramatically shortened *Drosophila* lifespan. We also confirmed that downregulation of 10 genes effectively suppressed proliferation of *Rbf1*-deficient tumor cells, even in the presence of additional strong oncogenic alterations.

### *RB1*-deficient human cancer cells are sensitive to chemical inhibitors of PIP5K1-, ubiquitin- and splicing-related pathways

**Fig 6:**
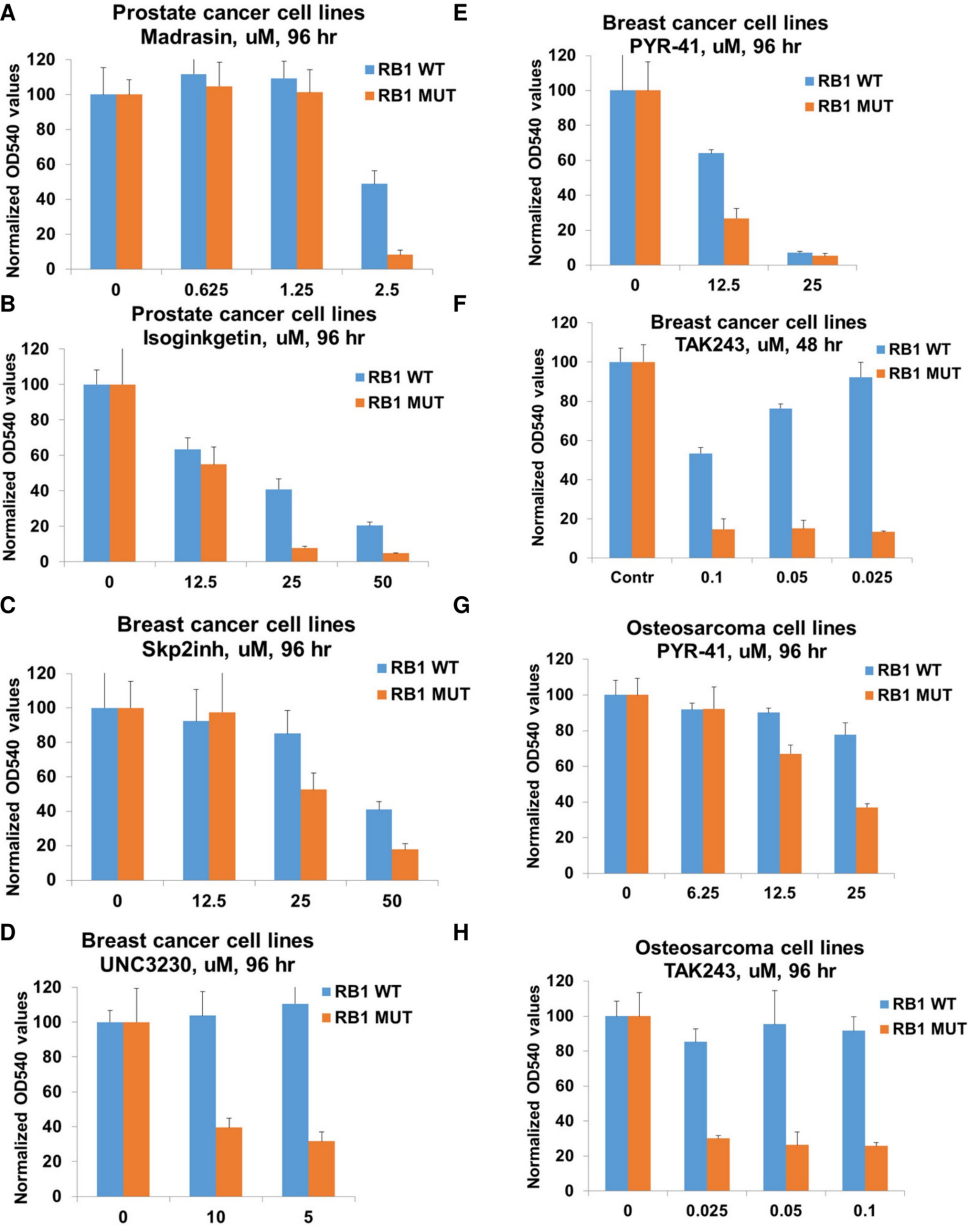
*RB1*-deficient cancer cells are sensitive to chemical inhibitors of PIP5K1-, ubiquitin- and splicing- related pathways. Proliferation of *RB1* wild-type and *RB1* mutant prostate cancer cells treated with 2.5, 1.25, or 0.625 uM of Madrasin **(A)** or 50, 25, or 12.5 uM of Isoginkgetin **(B)** for 96 hr (crystal violet staining). Data are shown as means ± SD. Proliferation of *RB1* wild-type and *RB1* mutant breast cancer cells treated with 50, 25, or 12.5 uM of Skp2 inhibitor **(C)**, 10 or 5 uM of UNC3230 **(D),** 25 or 12.5 uM of PYR-41 **(E)** or 0.1, 0.05, or 0.025 uM of TAK243 **(F)** for 96 hr (crystal violet staining). Data are shown as means ± SD. Proliferation of *RB1* wild-type and *RB1* mutant osteosarcoma cells treated with 25, 12.5 or 6.25 uM of PYR-41 **(G)** or 0.1, 0.05, or 0.025 uM of TAK243 **(H)** for 96 hr (crystal violet staining). Data are shown as means ± SD.

To further extend the utility of our findings we selected several small molecule inhibitors against some of the identified targets/pathways and tested them in three pairs of *RB1*-wt and -mutant human cancer cell lines. We used the following previously characterized pairs: *RB1*-wt PC3 and *RB1*-mutant DU145 prostate cancer cells, *RB1*-wt U2OS and *RB1*-mutant Saos-2 osteosarcoma cells, and *RB1*-wt MDA-MB-231 and *RB1*-mutant MDA-MB-468 triple negative breast cancer cells [11,54–58]. Because the GO analysis of the RB1 SL partners in our screen revealed that spliceosomal complex and ubiquitin-related pathways are among the top enriched groups (**Fig 1M)** and members of these groups were confirmed in follow-up analyses, we tested several chemical inhibitors against these pathways, as well as inhibitors of SKP2 and PIP5K1. To inhibit pre-mRNA splicing, we tested the natural product isoginkgetin, which inhibits splicing both *in vivo* and *in vitro* at micromolar concentrations [59], and madrasin [60]. Both pre-mRNA splicing inhibitors induced a dose-dependent response and had higher selectivity in *RB1*-deficient prostate cancer cells (**Fig 6A and 6B)**. SKP2 is probably one of the best characterized SL partners for RB1 and as SKP2 scored in line with our SL candidates, we tested a SKP2 inhibitor that disrupts the SKP2-SKP1 interaction and selectively prevents Skp2 complex-mediated substrate ubiquitination [61]. A SKP2 inhibitor inhibited proliferation of RB1-deficient breast cancer cells more efficiently than in *RB1*-proficient cells, and did so in a dose-dependent manner (**Fig 6C),** consistent with previous observations [13]. A PIP5K1 inhibitor, UNC3230, which has been identified as a chronic pain inhibitor [62], exhibited exceptional *RB1*-selectivity in breast cancer cells (**Fig 6D)**. Ubiquitination is catalyzed by the sequential action of ubiquitin-activating enzyme (E1), a ubiquitin-conjugating enzyme (E2), and a ubiquitin protein ligase (E3). Interestingly, two different inhibitors, PYR-41 and TAK-243, exhibited selective and dose-dependent responses in both breast cancer and osteosarcoma cell lines (**Fig 6E–6H).** PYR-41 has been identified as the first cell permeable ubiquitin-activating enzyme (E1) inhibitor [63]. TAK-243 (formerly known as MLN7243) is a potent inhibitor of ubiquitin-activating enzyme (E1) [64], which is currently being tested in cancer clinical trials (NCT03816319 and NCT02045095). Similar to UNC3230, TAK-243 exhibited exceptional selectivity in *RB1* mutant breast cancer and osteosarcoma cell lines, including a dramatic effect at nanomolar concentration range (**Fig 6F and 6H)**. Although, we found that two different splicing inhibitors (madrasin and isoginkgetin) and two different inhibitors of ubiquitin-activating enzyme (E1) (PYR-41 and TAK-243) demonstrated selective activity against RB1-deficient cells and complemented our genetic data, off-target activity may be also partially responsible for the observed effects. We also tested celastrol, the drug that we identified based on its inhibitory transcriptional effect on genes that function as *RB1* SL partners using CMap analysis (**Figs 4C and 4D** and S11B and S11C). A bioactive compound, celastrol is a pentacyclic triterpenoid with a broad range of biological activities and has been linked to antitumor activity [65]. Celastrol induced a dose-dependent response and had higher selectivity in *RB1*-deficient breast cancer cells (S12E Fig). We note that analysis of potential SL partners in human cancer cell lines revealed that the top 10 most responsive *RB1*-deficient human cancer cell lines for different hits were not from the same types of tumors. It is not surprising, then, that some inhibitors showed efficacy in one pair of cancer cell lines but not others. In conclusion, we identified several small molecule inhibitors that exhibit a selective response in *RB1*-deficient human cancer cell lines and suggest that TAK-243 might be easily repurposed for the treatment of *RB1*-deficient cancers in a clinical setting.

## Discussion

**Fig 7:**
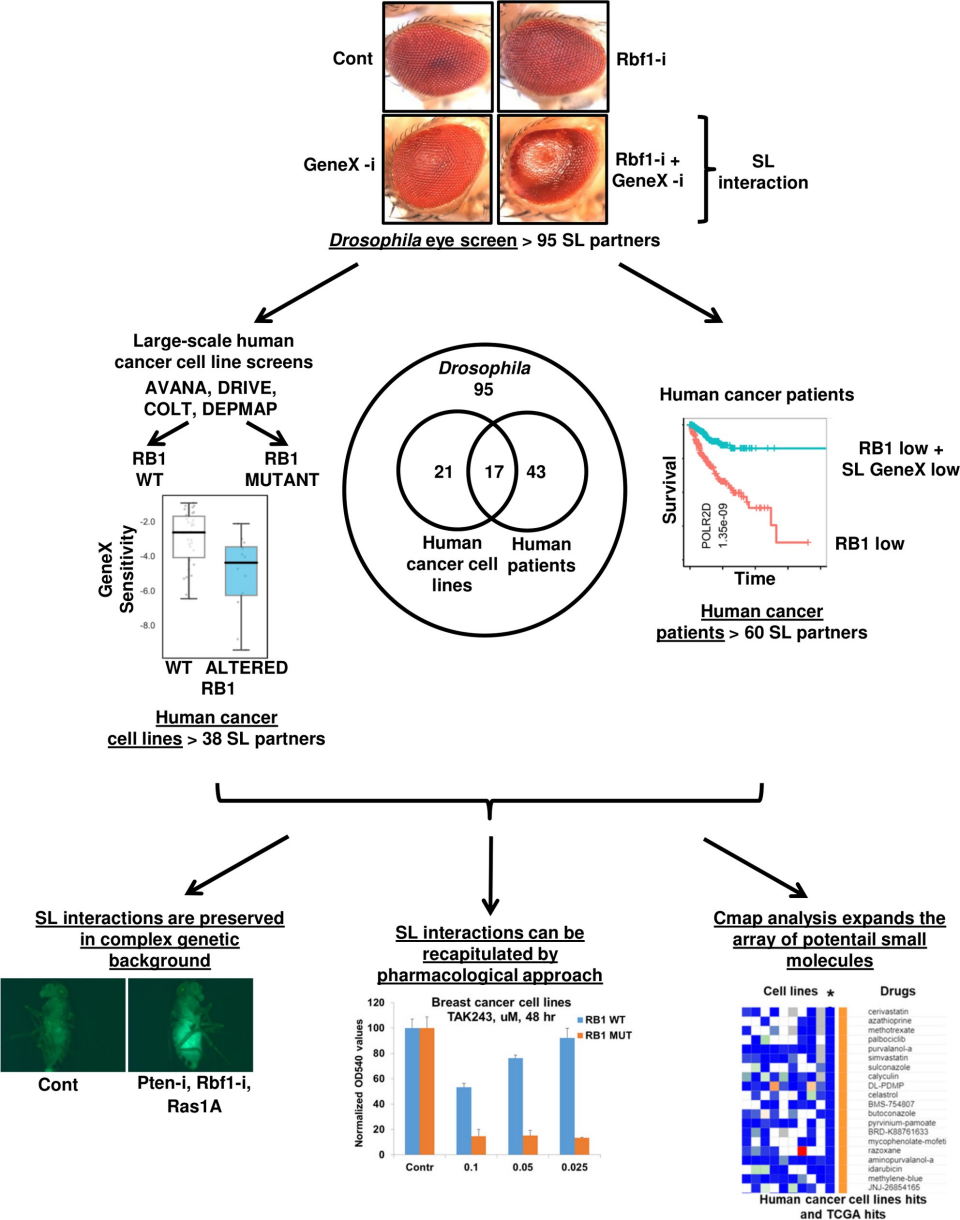
Overview of the findings presented in this study.

*RB1* is a tumor suppressor that is often mutated in multiple different tumors and finding new therapeutic targets to specifically kill *RB1*-deficient cells could potentially lead to a targeted therapy for these tumors. The Rb and E2F families are evolutionarily conserved in *Drosophila*. *Drosophila* has two Rb isoforms, *Rbf* and *Rbf2*, and two E2Fs, *dE2f1* and *dE2f2* [66–70]. We performed a genetic screen for SL partners of Rb in the *Drosophila* eye and then confirmed the validity of identified targets in human cancer cell lines and patient tumor samples. Furthermore, we tested several drugs against the identified targets/pathways that selectively killed *RB1*-deficient cells (**Fig 7**).

### *Drosophila* as a cancer model

*Drosophila* has been widely used for modeling cancer and development of cancer therapeutics [71–73]. Among the main advantages of *Drosophila* as a cancer model is the extraordinary evolutionary conservation of many of the basic processes of signal transduction mechanisms and transcriptional regulators between insects and vertebrates. Another advantage is the availability of sophisticated techniques for manipulating the fly genome and selection and analysis of mutant phenotypes. Recent advances in defining molecular portraits of different tumor subtypes, development of *in vivo* CRISPR/Cas9 tools, and use of *Drosophila* to model human cancers has allowed researchers to create *Drosophila* colorectal cancer models and to identify a personalized therapy via high-throughput screening of a library of FDA-approved drugs [74]. Indeed, the *Drosophila* hindgut has been used to model alterations in nine genes identified in a patient’s tumor. This model was used in a high-throughput screen that identified combination of trametinib and zoledronate as a potential cancer therapy. This led to a partial response in the patient [74]. In addition, the use of *Drosophila* for cancer modeling allows fast molecular profiling and toxicity evaluation that, for example, recently led to the identification of AD80 and AD81 as polypharmacological agents for RET-driven tumors [75]. However, one of the shortcomings of multiple current *Drosophila* cancer models is that they rely either on impaired development due to expression of oncogene or downregulation of tumor suppressor gene (eye or wing models) or on the overproliferation of stem cells (often intestinal stem cells), which do not completely recapitulate tumorigenesis in mammals and do not affect overall patient survival. Here, we created a novel *Drosophila* cancer model that causes dramatic and fast tumor overgrowth resulting in a significant decrease in fly overall survival. Moreover, this tumor overgrowth model can be monitored without fly dissection, allowing the system to be used in the future for high-throughput genetic and drug screens. Another advantage of using *Drosophila* in a search for potential drug targets is reduced genetic redundancy, as the presence of multiple homologs can mask the effects of loss-of-function experiments in mammalian genomes [76,77]. In our *Drosophila* eye screen, we identified several genes that exhibited a strong phenotype in *Rbf1*-deficient settings but some of these hits did not have a phenotype in human cancer cell lines or patient tumor samples. Although many factors could explain the lack of conservation in mammalian cells, one reason could be that mammals often have multiple orthologs of single *Drosophila* genes, and downregulation of one might be compensated by others. As an example, *Drosophila* gene *pyd* (polychaetoid), which scored in the eye screen, has three close orthologs in human cells: TJP1, TJP2, and TJP3. Neither of them scored in human cancer cell lines and in human cancer patients. Another reason might be the issue of low SL penetrance. Some gene pairs appear to be SL across multiple genetic backgrounds, but others are highly context-dependent [78]. A potential solution for this could be to use pharmacological inhibitors that target all close orthologs or combinatorial gene perturbation screens [79].

### RB1 therapeutic targets

One of the best-characterized SL interactions for the loss of *RB1* is *SKP2*. Skp2 forms a cullin-RING E3 ubiquitin ligase complex with cullin-1 and Skp1 to promote p21 and p27 degradation in S phase [80,81]. Inactivation of *Skp2* completely prevents spontaneous tumorigenesis in *Rb1* heterozygous mice. *RB1*-deficient human retinoblastoma cells undergo apoptosis after *Skp2* knockdown via the activation of p27 [12]. *Skp2* deletion also blocks *pRb*, *p53* double-deficient pituitary and prostate tumorigenesis [82]. In agreement with this, *SKP2* scored as a SL partner for *RB1*-deficient cells in both cancer cell line analysis and human cancer patients. In addition to *SKP2*, there were several other potential targets in *RB1*-deficient cells identified: *PPWD1/KIAA0073* [9], *TSC2* [10,11], *Dicer1* [14], *NUP88*, *NUP214*, *TAF1* [13], and *MED4* [15]. In addition, Aurora A kinase inhibitor LY3295668 [16], Aurora B kinase inhibitor AZD2811 [17], the mitotic inhibitors Taxol and STLC [18], and a SKP2 inhibitor [13] were all shown to be more selective against *RB1*-deficient cancer cells. Many of these genes and chemical inhibitor targets scored as SL partners for *RB1* loss in either the human cancer cell line analysis or in human cancer samples, further confirming the validity of our approach. In addition, the fact that many of these interactions were also recovered in the *Drosophila* eye further points out on the conservation of *RB1-*deficient targets between *Drosophila* and mammals. However, we believe that our approach is superior to the previous studies, because we were able to define SL interactions that were conserved in *Drosophila* and Human. Also of note is that previous parallel *in vivo* and *in vitro* screens in glioblastoma patient-derived cells revealed very little overlap between hits that caused cell depletion *in vitro* versus *in vivo* [83]. This potentially explains why the majority of identified drugs have never entered clinic use or failed in clinical trials. What is different about our approach is that our identified SL targets show efficacy in different species and both *in vitro* and *in vivo*. We propose that genes that scored at all steps of our pipeline (Subset 3 at **S3 Table**) are the most attractive targets for treatment of human cancers with *RB1*-deficiency and more likely to kill *RB1*-deficient cells in highly heterogeneous tumor cells. Moreover, we used three pairs of cancer cell lines with intact or mutant *RB1* and further demonstrated that madrasin, isoginkgetin, UNC3230, PYR-41, a Skp2-inhibitor, and TAK243 exhibited selective effects against *RB1*-deficient cells, with UNC3230 and TAK243 exhibiting the most dramatic therapeutic window between control and *RB1*-deficient cells. Notably, as any cancer might develop resistance against a particular drug, having multiple pharmacological targets opens wide possibilities for combinatorial trials. One caveat of this study is that we compared SL interactions in different organs/tissues using different readouts (development of the fly eye, loss of viability in cultured cells, and improved survival in cancer patients). Based on this, we do not state that the candidates that did not pass through the additional screens are not valid targets in *RB1*-deficient cells, we only state that the candidates that passed through all filters would more likely kill *RB1*-deficient cells in highly heterogeneous tumor cells.

### Functional interaction between RB1, PTEN, and RAS

Accumulation of additional mutations is an obligate event that leads to tumor progression, promotes metastasis, and contributes to drug resistance. We chose three signaling pathways that are known to cooperate in driving tumor progression in a diverse set of mouse cancer models, and members of these pathways are co-mutated in subsets of human cancer samples [47,48]. Mutations in the *PTEN*, *TP53*, and *RB1* signaling pathways are obligate events in the pathogenesis of human glioblastomas. Their combined inactivation accelerated the generation of high-grade astrocytomas in the adult mouse brain [84]. Co-deletion of *RB1* and *PTEN* in mouse osteoprogenitor cells promoted the formation of adipogenic tumors [85]. Co-deletion of *RB1*, *PTEN*, and *Rbl1 (p107)* in mouse retinal progenitor cells caused fully penetrant bilateral retinoblastomas [86]. Prostate-specific inactivation of the pRb family proteins (Rb/p107/p130) via SV40 large T antigen expression in combination with *Pten* hemizygosity accelerated development of prostate adenocarcinomas [87]. Similarly, *Rb1* loss promoted metastasis of prostate adenocarcinoma initiated by *Pten* mutation [88]. p16INK4A (encoded by CDKN2A) inhibited the cyclin D-CDK4/6 interactions and prevented phosphorylation of RB1, which blocks G1 to S-phase progression [89]. Melanocyte-specific loss of p16INK4a and activation of K-Ras promoted the development of melanomas [90]; B-cell specific inactivation of CDKN2A and activation of K-Ras promoted the development of highly aggressive B-cell acute lymphoblastic leukemia [91]; and alveolar-specific inactivation of CDKN2A and activation of K-Ras promotes development of lung adenocarcinomas [92]. We recapitulated these functional interactions among p16INK4a/RB1, Pten, and Ras oncogenic pathways, resulting in a highly aggressive tumor phenotype in our model that will be useful for high-throughput screens, for example done in parallel with mouse studies. We further tested 11 genes identified in our screen, as well as *SKP2* and *E2F*. Remarkably, despite the very aggressive tumor phenotype in our model, downregulation of 10 of 11 genes was still able to efficiently suppress proliferation of tumor cells while only moderately affecting control cells. Moreover, downregulation of several of these genes also significantly rescued the shortened lifespan of flies with the aggressive tumor growth without detrimental effect on the lifespan of control flies. There could be high value in testing the drugs we found to be effective in *RB1*-deficient human cancer cell lines in the mouse models mentioned above.

In summary, through a systematic multi-pronged approach, we identified several high-confidence, evolutionarily conserved, novel targets of *RB1-*deficient cells that may be further adapted for the treatment of human tumors.

## Material and methods

### Fly stocks, genetics

To assess eye phenotype, RNAi lines were crossed to the *GMR-GAL4* (II) driver line (Perrimon lab collection) at 25°C. For measuring luciferase and imaging GFP in intestine stem cells, *Gypsy-UAS-Luc@attp3; esg-Gal4*, *Gal80TS*, *UAS-GFP* stock (Perrimon lab collection) was crossed to RNAi lines at 18°C and switched to 29°C after eclosion for 7 days. The following RNAi lines were used: *white* RNAi (HMS00017); *GFP* RNAi (HMS00314); *Rbf1* RNAi (HMS03004), *Pten* RNAi (HMS00044), *Ran* RNAi (HMS02885), *SkpA* RNAi (HMS00791), *Cdc27* RNAi (HMC03814), *Cdc27* RNAi (HM04024), *Uba1* RNAi (JF01977), *Uba1* RNAi (HMS05878), *eIF4AIII* RNAi (HMS00442), *eIF4AIII* RNAi (HMS00696), *PIPK59B* RNAi (HMC05328), *PIPK59B* RNAi (HMJ23741), Sktl RNAi (JF02796), *Sce* RNAi (JF01396), *Sce* RNAi (HMS05745), SmF RNAi (JF02276), U2af50 RNAi (JF02693), U2af50 RNAi (HMC03810), U2af50 RNAi (HMJ23059), *CycA* RNAi (HMJ22195), *CycA* RNAi (JF02472), *Prosalpha7* RNAi (HMS00068), *Prosalpha7* RNAi (HMS05751), *Ada2a/Rpb4* RNAi (HMC05117), *Ada2a/Rpb4* RNAi (HMJ03126), *E2F* RNAi (HMS01541), *Skp2* RNAi (HMS00116).

### Eye pictures

Adult fly eyes were imaged in different focal planes using a SPOT RT3 camera (Model 25.4). CombineZP, a freely available software package was used for focus stacking. GNU Image Manipulation Program (GIMP) was used for image processing.

### Luciferase measurements

For luciferase measurements, four female flies were ground in 80 μL of Glo lysis buffer (Promega, E2661) containing 2 mM trypsin inhibitor Benzamidine (Sigma-Aldrich, B6506) and a protease inhibitor cocktail tablet (Roche, 4693159001). Ground flies were centrifuged at 12000 rpm 5 min a 4°C. 30 μL of lysates were transferred into 96-well opaque white plates and mixed with 30 μL of Steady Glo Luciferase assay kit (Promega, E2510). Luciferase activity was measured at Microplate Reader Spectramax Paradigm (Harvard Medical School DRSC facility).

### Cell lines

All cells were cultured in DMEM supplemented with 10% FBS, 100 μg/mL penicillin and 100 μg/mL streptomycin. All drugs were dissolved in DMSO. Madrasin (SML1409), Isoginkgetin (416154), Skp2 inhibitor (506305), PYR-41 (N2915) were ordered from Sigma-Aldrich, UNC 3230 (5271) from Tocris, TAK-243/MLN7243 (CT-M7243) from ChemieTek, celastrol from Cayman Chemical Company.

### Crystal violet staining

Cells were plated into 96-well plates (1000–1500 cells/well). After treatment, cells were fixed with 10% formalin for 5 minutes, stained with 0.05% crystal violet in distilled water for 30 minutes, washed 2 times with tap water, and drained. Crystal violet was solubilized with 100 ulof methanol and the plate was read with a BioTek plate reader (OD 540).

### qRT-PCR

Total RNA was extracted with the TRIzol reagent (Life Technologies), followed by DNase digestion using RQ1 RNase-Free DNase (Promega). Total RNA was reverse transcribed with the iScript cDNA synthesis kit (Bio-Rad). qRT-PCR was performed with the iQ SYBR Green Supermix (Bio-Rad) and a CFX96 Real- Time PCR Detection System (Bio-Rad). *RpL32* and *alpha-Tubulin 84B* were used as a normalization reference. Relative quantitation of mRNA levels was determined with the comparative C_T_ method. Primers for qRT-PCR were designed in accordance to [93].

### Gut staining

Female flies were dissected in cold PBS; fixed in 4% paraformaldehyde in PBS for 30 minutes; rinsed three times in PBS; blocked in PBS containing 5% goat serum and 0.5% Triton for 1 hour; stained with primary antibodies overnight at 4°C, washed three times in PBS containing 0.1% Triton; stained with secondary antibodies 2 hours at RT; washed three times in PBS containing 0.1% Triton; and mounted in vectashield containing DAPI.

### Confocal imaging

The guts were imaged with a Nikon Ti2 inverted microscope with W1 Yokogawa Spinning disk with 50 um pinhole disk at the MicRoN facility at Harvard Medical School (https://micron.hms.harvard.edu/equipment/garfunkel). The maximum projection image was generated using the freely available Fiji software.

### Bioinformatics analysis

We used the Gene List Annotation for Drosophila (GLAD) resource (http://www.flyrnai.org/tools/glad/web/) to perform gene set enrichment analysis [94]. The over-represented gene groups were illustrated using bar graph based on the negative log10 of the p values. The fly networks were built using the fly protein-protein interaction data from MIST release5 [34] and the network illustration was done using Cytoscape vs3.2.0 [95]. Fly hits were mapped to human orthologous using DIOPT [96] and human network was built using human protein-protein interaction data with the high or moderate rank from MIST release5. The complex analysis was done based on the complex annotation from COMPLEAT database [97].

### Analysis of loss-of-function screens in human cancer cell lines

Putative *RB1* dependencies were identified for the DRIVE, DEPMAP, and AVANA datasets in a similar fashion to [98]. DEPMAP (DEMETER V2) and AVANA (18Q4) datasets were obtained from the Depmap portal. The DRIVE dataset was obtained from the authors [19]. Associations between *RB1* status and gene sensitivity scores were identified in each screen using an ANOVA model that incorporated both tissue type and *RB1* status as covariates. In contrast to [98], which only analyzed a set of ‘selectively lethal genes’, associations were evaluated for all of the human orthologs of hits from the *Drosophila* screen. A gene was considered to be an *RB1* dependency in these screens if there was a nominal association (p<0.05) between *RB1* status and sensitivity to that gene. The *RB1* dependencies for the COLT dataset [22] were obtained from [13], where breast cancer cell lines were annotated according to RB1 status and the siMem algorithm [22] was used to identify dependencies.

### Analysis of drug screens in human cancer cell lines

AUC values for selected compounds from the CTRP, GDSC, and PRISM datasets were obtained from depmap.org [38,39,99,100]. For PRISM, only the data from the secondary screens were analyzed. Associations between drug sensitivity scores and *RB1* status were evaluated using the same linear model used to evaluate the loss-of-function screens. Drug association p-values were corrected for multiple-hypothesis testing using the Benjamini and Hochberg false-discovery rate [101]. Associations were considered significant at an FDR < 10%.

### Survival analysis

To test the clinical relevance of *Drosophila* synthetic lethal (SL) pairs of *RB1* in human cancer patients, we adapted our previously published pipeline called SPAGEfinder [8] which identifies the pairwise gene expression states that are significantly associated with the survival of cancer patients. Briefly, in SPAGEfinder, the expression of each gene across tumor samples is divided into 3 activity states namely; low, medium, and high, resulting in a total of 9 possible joint activity states between two genes called interaction-bins. We were interested only in the SL interactions of *RB1* and we focused on just one interaction bin where the expression of both the genes is downregulated in cancer patients (Bin1 in Fig 3A), and one of the genes is *RB1*. We compared the overall survival of patients in Bin1 (both genes downregulated) against those in Bin7 (only *RB1* is downregulated). Gene expression and clinical data were taken from TCGA for lung, breast and prostate cancers as RB1 is frequently mutated in these cancer types.

## Supporting information

S1 Fig *Drosophila* eye pictures of the following genotypes: *GMR-Gal4*> RNAi X from screen library and *GMR-Gal4*> *Rbf1-i*, RNAi X. It should be noted that the first two control pictures *GMR-Gal4*> Contr RNAi and *GMR-Gal4*> *Rbf1-i* are similar at all supplemental figures and similar to Fig 1B and 1C. They are added for easier comparison of phenotypes between different figures.
(TIF)

S2 Fig *Drosophila* eye pictures of the following genotypes: *GMR-Gal4*> RNAi X from screen library and *GMR-Gal4*> *Rbf1-i*, RNAi X. It should be noted that the first two control pictures *GMR-Gal4*> Contr RNAi and *GMR-Gal4*> *Rbf1-i* are similar at all supplemental figures and similar to Fig 1B and 1C. They are added for easier comparison of phenotypes between different figures.
(TIF)

S3 Fig *Drosophila* eye pictures of the following genotypes: *GMR-Gal4*> RNAi X from screen library and *GMR-Gal4*> *Rbf1-i*, RNAi X. It should be noted that the first two control pictures *GMR-Gal4*> Contr RNAi and *GMR-Gal4*> *Rbf1-i* are similar at all supplemental figures and similar to Fig 1B and 1C. They are added for easier comparison of phenotypes between different figures.
(TIF)

S4 Fig *Drosophila* eye pictures of the following genotypes: *GMR-Gal4*> RNAi X from screen library and *GMR-Gal4*> *Rbf1-i*, RNAi X. It should be noted that the first two control pictures *GMR-Gal4*> Contr RNAi and *GMR-Gal4*> *Rbf1-i* are similar at all supplemental figures and similar to Fig 1B and 1C. They are added for easier comparison of phenotypes between different figures.
(TIF)

S5 Fig *Drosophila* eye pictures of the following genotypes: *GMR-Gal4*> RNAi X from screen library and *GMR-Gal4*> *Rbf1-i*, RNAi X. It should be noted that the first two control pictures *GMR-Gal4*> Contr RNAi and *GMR-Gal4*> *Rbf1-i* are similar at all supplemental figures and similar to Fig 1B and 1C. They are added for easier comparison of phenotypes between different figures.
(TIF)

S6 Fig *Drosophila* eye pictures of the following genotypes: *GMR-Gal4*> RNAi X from screen library and *GMR-Gal4*> *Rbf1-i*, RNAi X. It should be noted that the first two control pictures *GMR-Gal4*> Contr RNAi and *GMR-Gal4*> *Rbf1-i* are similar at all supplemental figures and similar to Fig 1B and 1C. They are added for easier comparison of phenotypes between different figures.
(TIF)

S7 Fig *Drosophila* eye pictures of the following genotypes: *GMR-Gal4*> RNAi X from screen library and *GMR-Gal4*> *Rbf1-i*, RNAi X. It should be noted that the first two control pictures *GMR-Gal4*> Contr RNAi and *GMR-Gal4*> *Rbf1-i* are similar at all supplemental figures and similar to Fig 1B and 1C. They are added for easier comparison of phenotypes between different figures.
(TIF)

S8 Fig *Drosophila* eye pictures of the following genotypes: *GMR-Gal4*> RNAi X from screen library and *GMR-Gal4*> *Rbf1-i*, RNAi X. It should be noted that the first two control pictures *GMR-Gal4*> Contr RNAi and *GMR-Gal4*> *Rbf1-i* are similar at all supplemental figures and similar to Fig 1B and 1C. They are added for easier comparison of phenotypes between different figures.
(TIF)

S9 Fig Kaplan Meier’s survival curves along with corrected p-values for 12 genes that showed SL with RB1 in cancer patients and in one of human cancer cell lines screens (A). Kaplan Meier’s survival curves along with corrected p-values for 11 genes positive controls from literature (B). Blue line–levels of both genes (RB1 and outlined gene) are low, red line–only RB1 level is low.
(TIF)

S10 Fig The overall survival of the TCGA cancer patients by comparing the patients with low activity of both genes against the low activity of only *CDKN2A* in lung, breast, and prostate cancers patients. The overall survival of the TCGA cancer patients by comparing the patients with high activity of *CCND1* (**B**), *CDK4* (**C**), or *CDK6* (**D**) and low activity of genes from the screen against the high activity of only CCND1 (**B**), CDK4 (**C**), or CDK6 (**D**) in lung, breast, and prostate cancers patients. Box plot comparing the relative hazard computed using log-rank test for different gene sets.
(TIF)

S11 Fig The distribution of connectivity for 1000 lists of randomly selected 95 genes from all the genes screened (**A**). Top 20 drugs from the connectivity map analysis that scored from the analysis of subnetwork 2 that contains proteins that also scored as SL partners in TCGA human cancer patients (**B**). Top 20 drugs from the connectivity map analysis of proteins that scored as SL partners in both human cancer cell lines and TCGA human cancer patients (**C**).
(TIF)

S12 Fig Lifespan analysis of *UAS-Luciferase*, *esg-Gal4*, *tubulinGal80ts*, *UAS-GFP* lines containing either control RNAi (control) or combination of *Rbf1* RNAi, *Pten* RNAi, *Ras1A* overexpression and crossed to either control RNAi or RNAi against Uba1 (**A**), Cdc27 (**B**), Ada2a/Rpb4 (**C**), or eIF4AIII (**D**). Note that the lifespans of control RNAi flies are the same between different panels. Proliferation of *RB1* wild-type and *RB1* mutant breast cancer cells treated with 1, 0.5, 0.25, or 0.125 uM of celastrol for 96 hr (crystal violet staining). Data are shown as means ± SD. (**E**).
(TIF)

S1 Table The information on RNAi lines that have been used for the *Drosophila* eye screen. (XLSX)

S2 Table The information on 95 hits from the *Drosophila* eye screen, their human orthologs; the effects of the depletion of their human orthologs on the proliferation of human cancer cell lines from the Marcotte/COLT, DRIVE, AVANA, and DEPMAP projects (0 –no effect, 1 –significant effect); and results from SPAGEfinder on the interactions of their human orthologs and RB1 in cancer samples from human patients. (XLSX)

S3 Table Subsets of genes that have been used for the connectivity map analysis and top 20 drugs retrieved for each subset. (XLSX)

S4 Table Drug sensitivity profiles for 29 compounds from the Connectivity Map (CMap) analysis from three different drug screening resources: CTRP, GDSC, and PRISM. (CSV)

## References

[1] Priestley P, Baber J, Lolkema MP, Steeghs N, de Bruijn E, Shale C, et al. Pan-cancer whole-genome analyses of metastatic solid tumours. Nature. 2019;575(7781):210–6. Epub 2019/10/28. 10.1038/s41586-019-1689-y 31645765 PMC6872491

[2] Sherr CJ, McCormick F. The RB and p53 pathways in cancer. Cancer Cell 2002;2(2):103–12. Epub 2002/09/03. 10.1016/s1535-6108(02)00102-2 . 12204530

[3] Dyson NJ. RB1: a prototype tumor suppressor and an enigma. Genes & development. 2016;30(13):1492–502. Epub 2016/07/13. 10.1101/gad.282145.116 27401552 PMC4949322

[4] Gordon GM, Du W. Conserved RB functions in development and tumor suppression. Protein Cell. 2011;2(11):864–78. Epub 2011/12/20. 10.1007/s13238-011-1117-z 22180086 PMC3271014

[5] Velez-Cruz R, Johnson DG. The Retinoblastoma (RB) Tumor Suppressor: Pushing Back against Genome Instability on Multiple Fronts. Int J Mol Sci. 2017;18(8). Epub 2017/08/17. 10.3390/ijms18081776 28812991 PMC5578165

[6] Lee JS, Das A, Jerby-Arnon L, Arafeh R, Auslander N, Davidson M, et al. Harnessing synthetic lethality to predict the response to cancer treatment. Nature communications. 2018;9(1):2546. Epub 2018/07/01. 10.1038/s41467-018-04647-1 29959327 PMC6026173

[7] O’Neil NJ, Bailey ML, Hieter P. Synthetic lethality and cancer. Nat Rev Genet 2017;18(10):613–23. Epub 2017/06/27. 10.1038/nrg.2017.47 . 28649135

[8] Magen A, Das Sahu A, Lee JS, Sharmin M, Lugo A, Gutkind JS, et al. Beyond Synthetic Lethality: Charting the Landscape of Pairwise Gene Expression States Associated with Survival in Cancer. Cell reports. 2019;28(4):938–48 e6. Epub 2019/07/25. 10.1016/j.celrep.2019.06.067 . 31340155 PMC8261641

[9] Edgar KA, Belvin M, Parks AL, Whittaker K, Mahoney MB, Nicoll M, et al. Synthetic lethality of retinoblastoma mutant cells in the Drosophila eye by mutation of a novel peptidyl prolyl isomerase gene. Genetics. 2005;170(1):161–71. Epub 2005/03/04. 10.1534/genetics.104.036343 15744054 PMC1449713

[10] Gordon GM, Du W. Targeting Rb inactivation in cancers by synthetic lethality. Am J Cancer Res. 2011;1(6):773–86. Epub 2011/08/05. 21814623 PMC3147291

[11] Li B, Gordon GM, Du CH, Xu J, Du W. Specific killing of Rb mutant cancer cells by inactivating TSC2. Cancer cell. 2010;17(5):469–80. Epub 2010/05/19. 10.1016/j.ccr.2010.03.019 20478529 PMC2873973

[12] Wang H, Bauzon F, Ji P, Xu X, Sun D, Locker J, et al. Skp2 is required for survival of aberrantly proliferating Rb1-deficient cells and for tumorigenesis in Rb1+/- mice. Nat Genet. 2010;42(1):83–8. Epub 2009/12/08. 10.1038/ng.498 19966802 PMC2990528

[13] Brough R, Gulati A, Haider S, Kumar R, Campbell J, Knudsen E, et al. Identification of highly penetrant Rb-related synthetic lethal interactions in triple negative breast cancer. Oncogene. 2018;37(43):5701–18. Epub 2018/06/20. 10.1038/s41388-018-0368-z 29915391 PMC6202330

[14] Nittner D, Lambertz I, Clermont F, Mestdagh P, Kohler C, Nielsen SJ, et al. Synthetic lethality between Rb, p53 and Dicer or miR-17-92 in retinal progenitors suppresses retinoblastoma formation. Nature cell biology. 2012;14(9):958–65. Epub 2012/08/07. 10.1038/ncb2556 . 22864477

[15] Dehainault C, Garancher A, Castera L, Cassoux N, Aerts I, Doz F, et al. The survival gene MED4 explains low penetrance retinoblastoma in patients with large RB1 deletion. Hum Mol Genet. 2014;23(19):5243–50. Epub 2014/05/27. 10.1093/hmg/ddu245 . 24858910

[16] Gong X, Du J, Parsons SH, Merzoug FF, Webster Y, Iversen PW, et al. Aurora A Kinase Inhibition Is Synthetic Lethal with Loss of the RB1 Tumor Suppressor Gene. Cancer Discov. 2019;9(2):248–63. Epub 2018/10/31. 10.1158/2159-8290.CD-18-0469 . 30373917

[17] Oser MG, Fonseca R, Chakraborty AA, Brough R, Spektor A, Jennings RB, et al. Cells Lacking the RB1 Tumor Suppressor Gene Are Hyperdependent on Aurora B Kinase for Survival. Cancer Discov. 2019;9(2):230–47. Epub 2018/10/31. 10.1158/2159-8290.CD-18-0389 30373918 PMC6368871

[18] Zhao J, Zhang Z, Liao Y, Du W. Mutation of the retinoblastoma tumor suppressor gene sensitizes cancers to mitotic inhibitor induced cell death. Am J Cancer Res. 2014;4(1):42–52. Epub 2014/02/01. 24482737 PMC3902231

[19] McDonald ER, de Weck A, Schlabach MR, Billy E, Mavrakis KJ, Hoffman GR, et al. Project DRIVE: A Compendium of Cancer Dependencies and Synthetic Lethal Relationships Uncovered by Large-Scale, Deep RNAi Screening. Cell. 2017;170(3):577–92 e10. Epub 2017/07/29. 10.1016/j.cell.2017.07.005 . 28753431

[20] Meyers RM, Bryan JG, McFarland JM, Weir BA, Sizemore AE, Xu H, et al. Computational correction of copy number effect improves specificity of CRISPR-Cas9 essentiality screens in cancer cells. Nat Genet. 2017;49(12):1779–84. Epub 2017/10/31. 10.1038/ng.3984 29083409 PMC5709193

[21] Tsherniak A, Vazquez F, Montgomery PG, Weir BA, Kryukov G, Cowley GS, et al. Defining a Cancer Dependency Map. Cell. 2017;170(3):564–76 e16. Epub 2017/07/29. 10.1016/j.cell.2017.06.010 28753430 PMC5667678

[22] Marcotte R, Sayad A, Brown KR, Sanchez-Garcia F, Reimand J, Haider M, et al. Functional Genomic Landscape of Human Breast Cancer Drivers, Vulnerabilities, and Resistance. Cell. 2016;164(1–2):293–309. Epub 2016/01/16. 10.1016/j.cell.2015.11.062 26771497 PMC4724865

[23] Nicolay BN, Gameiro PA, Tschop K, Korenjak M, Heilmann AM, Asara JM, et al. Loss of RBF1 changes glutamine catabolism. Genes & development. 2013;27(2):182–96. Epub 2013/01/17. 10.1101/gad.206227.112 23322302 PMC3566311

[24] Freeman M. Reiterative use of the EGF receptor triggers differentiation of all cell types in the Drosophila eye. Cell 1996;87(4):651–60. Epub 1996/11/15. 10.1016/s0092-8674(00)81385-9 . 8929534

[25] Nicolay BN, Bayarmagnai B, Moon NS, Benevolenskaya EV, Frolov MV. Combined inactivation of pRB and hippo pathways induces dedifferentiation in the Drosophila retina. PLoS Genet. 2010;6(4):e1000918. Epub 2010/04/28. 10.1371/journal.pgen.1000918 20421993 PMC2858677

[26] Vidal M, Wells S, Ryan A, Cagan R. ZD6474 suppresses oncogenic RET isoforms in a Drosophila model for type 2 multiple endocrine neoplasia syndromes and papillary thyroid carcinoma. Cancer Res 2005;65(9):3538–41. Epub 2005/05/04. 10.1158/0008-5472.CAN-04-4561 . 15867345

[27] Bach EA, Vincent S, Zeidler MP, Perrimon N. A sensitized genetic screen to identify novel regulators and components of the Drosophila janus kinase/signal transducer and activator of transcription pathway. Genetics. 2003;165(3):1149–66. Epub 2003/12/12. 14668372 10.1093/genetics/165.3.1149 PMC1462825

[28] Khurana V, Lu Y, Steinhilb ML, Oldham S, Shulman JM, Feany MB. TOR-mediated cell-cycle activation causes neurodegeneration in a Drosophila tauopathy model. Curr Biol 2006;16(3):230–41. Epub 2006/02/08. 10.1016/j.cub.2005.12.042 . 16461276

[29] Hanahan D, Weinberg RA. Hallmarks of cancer: the next generation. Cell 2011;144(5):646–74. Epub 2011/03/08. 10.1016/j.cell.2011.02.013 . 21376230

[30] Costanzo M, VanderSluis B, Koch EN, Baryshnikova A, Pons C, Tan G, et al. A global genetic interaction network maps a wiring diagram of cellular function. Science (New York, NY. 2016;353(6306). Epub 2016/10/07. 10.1126/science.aaf1420 27708008 PMC5661885

[31] Kelley R, Ideker T. Systematic interpretation of genetic interactions using protein networks. Nat Biotechnol. 2005;23(5):561–6. Epub 2005/05/07. 10.1038/nbt1096 15877074 PMC2814446

[32] Collins SR, Miller KM, Maas NL, Roguev A, Fillingham J, Chu CS, et al. Functional dissection of protein complexes involved in yeast chromosome biology using a genetic interaction map. Nature. 2007;446(7137):806–10. Epub 2007/02/23. 10.1038/nature05649 . 17314980

[33] Kaplow IM, Singh R, Friedman A, Bakal C, Perrimon N, Berger B. RNAiCut: automated detection of significant genes from functional genomic screens. Nat Methods 2009;6(7):476–7. Epub 2009/07/01. 10.1038/nmeth0709-476 . 19564846

[34] Hu Y, Vinayagam A, Nand A, Comjean A, Chung V, Hao T, et al. Molecular Interaction Search Tool (MIST): an integrated resource for mining gene and protein interaction data. Nucleic acids research. 2018;46(D1):D567–D74. Epub 2017/11/21. 10.1093/nar/gkx1116 29155944 PMC5753374

[35] Subramanian A, Narayan R, Corsello SM, Peck DD, Natoli TE, Lu X, et al. A Next Generation Connectivity Map: L1000 Platform and the First 1,000,000 Profiles. Cell. 2017;171(6):1437–52 e17. Epub 2017/12/02. 10.1016/j.cell.2017.10.049 29195078 PMC5990023

[36] Lamb J, Crawford ED, Peck D, Modell JW, Blat IC, Wrobel MJ, et al. The Connectivity Map: using gene-expression signatures to connect small molecules, genes, and disease. Science (New York, NY. 2006;313(5795):1929–35. Epub 2006/09/30. 10.1126/science.1132939 . 17008526

[37] Corsello SM, Nagari RT, Spangler RD, Rossen J, Kocak M, Bryan JG, et al. Discovering the anticancer potential of non-oncology drugs by systematic viability profiling. Nature Cancer. 2020;1:235–48. 10.1038/s43018-019-0018-6 32613204 PMC7328899

[38] Iorio F, Knijnenburg TA, Vis DJ, Bignell GR, Menden MP, Schubert M, et al. A Landscape of Pharmacogenomic Interactions in Cancer. Cell. 2016;166(3):740–54. Epub 2016/07/12. 10.1016/j.cell.2016.06.017 27397505 PMC4967469

[39] Seashore-Ludlow B, Rees MG, Cheah JH, Cokol M, Price EV, Coletti ME, et al. Harnessing Connectivity in a Large-Scale Small-Molecule Sensitivity Dataset. Cancer Discov. 2015;5(11):1210–23. Epub 2015/10/21. 10.1158/2159-8290.CD-15-0235 26482930 PMC4631646

[40] Qaddoumi I, Billups CA, Tagen M, Stewart CF, Wu J, Helton K, et al. Topotecan and vincristine combination is effective against advanced bilateral intraocular retinoblastoma and has manageable toxicity. Cancer. 2012;118(22):5663–70. Epub 2012/04/21. 10.1002/cncr.27563 22516936 PMC3413782

[41] Coussy F, El-Botty R, Chateau-Joubert S, Dahmani A, Montaudon E, Leboucher S, et al. BRCAness, SLFN11, and RB1 loss predict response to topoisomerase I inhibitors in triple-negative breast cancers. Sci Transl Med. 2020;12(531). Epub 2020/02/23. 10.1126/scitranslmed.aax2625 . 32075943 PMC8662740

[42] Xiao H, Goodrich DW. 10.1038/sj.onc.1208958 retinoblastoma tumor suppressor protein is required for efficient processing and repair of trapped topoisomerase II-DNA-cleavable complexes. Oncogene. 2005;24(55):8105–13. Epub 2005/08/11. PubMed Central PMCID: PMC2799250. 16091739 PMC2799250

[43] Sanchez-Vega F, Mina M, Armenia J, Chatila WK, Luna A, La KC, et al. Oncogenic Signaling Pathways in The Cancer Genome Atlas. Cell. 2018;173(2):321–37 e10. Epub 2018/04/07. 10.1016/j.cell.2018.03.035 29625050 PMC6070353

[44] Vasan N, Baselga J, Hyman DM. A view on drug resistance in cancer. Nature 2019;575(7782):299–309. Epub 2019/11/15. 10.1038/s41586-019-1730-1 . 31723286 PMC8008476

[45] Kuzmin E, VanderSluis B, Wang W, Tan G, Deshpande R, Chen Y, et al. Systematic analysis of complex genetic interactions. Science (New York, NY. 2018;360(6386). Epub 2018/04/21. 10.1126/science.aao1729 29674565 PMC6215713

[46] Lord CJ, Ashworth A. PARP inhibitors: Synthetic lethality in the clinic. Science (New York, NY. 2017;355(6330):1152–8. Epub 2017/03/18. 10.1126/science.aam7344 28302823 PMC6175050

[47] Cerami E, Gao J, Dogrusoz U, Gross BE, Sumer SO, Aksoy BA, et al. The cBio cancer genomics portal: an open platform for exploring multidimensional cancer genomics data. Cancer Discov. 2012;2(5):401–4. Epub 2012/05/17. 10.1158/2159-8290.CD-12-0095 22588877 PMC3956037

[48] Gao J, Aksoy BA, Dogrusoz U, Dresdner G, Gross B, Sumer SO, et al. Integrative analysis of complex cancer genomics and clinical profiles using the cBioPortal. Science signaling. 2013;6(269):pl1. Epub 2013/04/04. 10.1126/scisignal.2004088 23550210 PMC4160307

[49] Haley B, Foys B, Levine M. Vectors and parameters that enhance the efficacy of RNAi-mediated gene disruption in transgenic Drosophila. Proceedings of the National Academy of Sciences of the United States of America. 2010;107(25):11435–40. Epub 2010/06/11. 10.1073/pnas.1006689107 20534445 PMC2895090

[50] Lu X, Chou TB, Williams NG, Roberts T, Perrimon N. Control of cell fate determination by p21ras/Ras1, an essential component of torso signaling in Drosophila. Genes Dev 1993;7(4):621–32. Epub 1993/04/01. 10.1101/gad.7.4.621 . 8458578

[51] Gisselbrecht S, Skeath JB, Doe CQ, Michelson AM. heartless encodes a fibroblast growth factor receptor (DFR1/DFGF-R2) involved in the directional migration of early mesodermal cells in the Drosophila embryo. Genes Dev 1996;10(23):3003–17. Epub 1996/12/01. 10.1101/gad.10.23.3003 . 8957001

[52] Markstein M, Dettorre S, Cho J, Neumuller RA, Craig-Muller S, Perrimon N. Systematic screen of chemotherapeutics in Drosophila stem cell tumors. Proceedings of the National Academy of Sciences of the United States of America. 2014;111(12):4530–5. Epub 2014/03/13. 10.1073/pnas.1401160111 24616500 PMC3970492

[53] Biteau B, Karpac J, Supoyo S, Degennaro M, Lehmann R, Jasper H. Lifespan extension by preserving proliferative homeostasis in Drosophila. PLoS Genet. 2010;6(10):e1001159. Epub 2010/10/27. 10.1371/journal.pgen.1001159 20976250 PMC2954830

[54] Tan HL, Sood A, Rahimi HA, Wang W, Gupta N, Hicks J, et al. Rb loss is characteristic of prostatic small cell neuroendocrine carcinoma. Clin Cancer Res. 2014;20(4):890–903. Epub 2013/12/11. 10.1158/1078-0432.CCR-13-1982 24323898 PMC3931005

[55] Cook R, Zoumpoulidou G, Luczynski MT, Rieger S, Moquet J, Spanswick VJ, et al. Direct involvement of retinoblastoma family proteins in DNA repair by non-homologous end-joining. Cell reports. 2015;10(12):2006–18. Epub 2015/03/31. 10.1016/j.celrep.2015.02.059 25818292 PMC4386026

[56] Hellwinkel OJ, Muller J, Pollmann A, Kabisch H. Osteosarcoma cell lines display variable individual reactions on wildtype p53 and Rb tumour-suppressor transgenes. J Gene Med 2005;7(4):407–19. Epub 2004/11/13. 10.1002/jgm.684 . 15538723

[57] Jones RA, Robinson TJ, Liu JC, Shrestha M, Voisin V, Ju Y, et al. RB1 deficiency in triple-negative breast cancer induces mitochondrial protein translation. The Journal of clinical investigation. 2016;126(10):3739–57. Epub 2016/08/30. 10.1172/JCI81568 27571409 PMC5096803

[58] Robinson TJ, Liu JC, Vizeacoumar F, Sun T, Maclean N, Egan SE, et al. RB1 status in triple negative breast cancer cells dictates response to radiation treatment and selective therapeutic drugs. PLoS One. 2013;8(11):e78641. Epub 2013/11/23. 10.1371/journal.pone.0078641 24265703 PMC3827056

[59] O’Brien K, Matlin AJ, Lowell AM, Moore MJ. The biflavonoid isoginkgetin is a general inhibitor of Pre-mRNA splicing. The Journal of biological chemistry. 2008;283(48):33147–54. Epub 2008/10/02. 10.1074/jbc.M805556200 18826947 PMC2586251

[60] Pawellek A, McElroy S, Samatov T, Mitchell L, Woodland A, Ryder U, et al. Identification of small molecule inhibitors of pre-mRNA splicing. The Journal of biological chemistry. 2014;289(50):34683–98. Epub 2014/10/05. 10.1074/jbc.M114.590976 25281741 PMC4263873

[61] Chan CH, Morrow JK, Li CF, Gao Y, Jin G, Moten A, et al. Pharmacological inactivation of Skp2 SCF ubiquitin ligase restricts cancer stem cell traits and cancer progression. Cell. 2013;154(3):556–68. Epub 2013/08/06. 10.1016/j.cell.2013.06.048 23911321 PMC3845452

[62] Wright BD, Loo L, Street SE, Ma A, Taylor-Blake B, Stashko MA, et al. The lipid kinase PIP5K1C regulates pain signaling and sensitization. Neuron. 2014;82(4):836–47. Epub 2014/05/24. 10.1016/j.neuron.2014.04.006 24853942 PMC4074510

[63] Yang Y, Kitagaki J, Dai RM, Tsai YC, Lorick KL, Ludwig RL, et al. Inhibitors of ubiquitin-activating enzyme (E1), a new class of potential cancer therapeutics. Cancer research. 2007;67(19):9472–81. Epub 2007/10/03. 10.1158/0008-5472.CAN-07-0568 . 17909057

[64] Hyer ML, Milhollen MA, Ciavarri J, Fleming P, Traore T, Sappal D, et al. A small-molecule inhibitor of the ubiquitin activating enzyme for cancer treatment. Nat Med. 2018;24(2):186–93. Epub 2018/01/16. 10.1038/nm.4474 . 29334375

[65] Cascao R, Fonseca JE, Moita LF. Celastrol: A Spectrum of Treatment Opportunities in Chronic Diseases. Front Med (Lausanne). 2017;4:69. Epub 2017/07/01. 10.3389/fmed.2017.00069 28664158 PMC5471334

[66] Dynlacht BD, Brook A, Dembski M, Yenush L, Dyson N. DNA-binding and trans-activation properties of Drosophila E2F and DP proteins. Proceedings of the National Academy of Sciences of the United States of America. 1994;91(14):6359–63. Epub 1994/07/05. 10.1073/pnas.91.14.6359 8022787 PMC44201

[67] Du W, Vidal M, Xie JE, Dyson N. RBF, a novel RB-related gene that regulates E2F activity and interacts with cyclin E in Drosophila. Genes Dev 1996;10(10):1206–18. Epub 1996/05/15. 10.1101/gad.10.10.1206 . 8675008

[68] Stevaux O, Dimova D, Frolov MV, Taylor-Harding B, Morris E, Dyson N. Distinct mechanisms of E2F regulation by Drosophila RBF1 and RBF2. The EMBO journal. 2002;21(18):4927–37. Epub 2002/09/18. 10.1093/emboj/cdf501 12234932 PMC126297

[69] Sawado T, Yamaguchi M, Nishimoto Y, Ohno K, Sakaguchi K, Matsukage A. dE2F2, a novel E2F-family transcription factor in Drosophila melanogaster. Biochem Biophys Res Commun 1998;251(2):409–15. Epub 1998/10/30. 10.1006/bbrc.1998.9407 . 9792788

[70] Ohtani K, Nevins JR. Functional properties of a Drosophila homolog of the E2F1 gene. Molecular and cellular biology. 1994;14(3):1603–12. Epub 1994/03/01. 10.1128/mcb.14.3.1603 8114698 PMC358519

[71] Yadav AK, Srikrishna S, Gupta SC. Cancer Drug Development Using Drosophila as an in vivo Tool: From Bedside to Bench and Back. Trends Pharmacol Sci 2016;37(9):789–806. Epub 2016/06/15. 10.1016/j.tips.2016.05.010 . 27298020

[72] Gonzalez C. Drosophila melanogaster: a model and a tool to investigate malignancy and identify new therapeutics. Nature reviews 2013;13(3):172–83. Epub 2013/02/08. 10.1038/nrc3461 . 23388617

[73] Villegas SN. One hundred years of Drosophila cancer research: no longer in solitude. Disease models & mechanisms. 2019;12(4). Epub 2019/04/07. 10.1242/dmm.039032 30952627 PMC6505481

[74] Bangi E, Ang C, Smibert P, Uzilov AV, Teague AG, Antipin Y, et al. A personalized platform identifies trametinib plus zoledronate for a patient with KRAS-mutant metastatic colorectal cancer. Sci Adv. 2019;5(5):eaav6528. Epub 2019/05/28. 10.1126/sciadv.aav6528 31131321 PMC6531007

[75] Dar AC, Das TK, Shokat KM, Cagan RL. Chemical genetic discovery of targets and anti-targets for cancer polypharmacology. Nature. 2012;486(7401):80–4. Epub 2012/06/09. 10.1038/nature11127 22678283 PMC3703503

[76] De Kegel B, Ryan CJ. Paralog buffering contributes to the variable essentiality of genes in cancer cell lines. PLoS Genet. 2019;15(10):e1008466. Epub 2019/10/28. 10.1371/journal.pgen.1008466 31652272 PMC6834290

[77] Ewen-Campen B, Mohr SE, Hu Y, Perrimon N. Accessing the Phenotype Gap: Enabling Systematic Investigation of Paralog Functional Complexity with CRISPR. Dev Cell 2017;43(1):6–9. Epub 2017/10/11. 10.1016/j.devcel.2017.09.020 . 29017030

[78] Ryan CJ, Bajrami I, Lord CJ. Synthetic Lethality and Cancer—Penetrance as the Major Barrier. Trends Cancer 2018;4(10):671–83. Epub 2018/10/08. 10.1016/j.trecan.2018.08.003 . 30292351

[79] Gonatopoulos-Pournatzis T, Aregger M, Brown KR, Farhangmehr S, Braunschweig U, Ward HN, et al. Genetic interaction mapping and exon-resolution functional genomics with a hybrid Cas9-Cas12a platform. Nat Biotechnol. 2020;38(5):638–48. Epub 2020/04/07. 10.1038/s41587-020-0437-z . 32249828

[80] Bornstein G, Bloom J, Sitry-Shevah D, Nakayama K, Pagano M, Hershko A. Role of the SCFSkp2 ubiquitin ligase in the degradation of p21Cip1 in S phase. J Biol Chem 2003;278(28):25752–7. Epub 2003/05/06. 10.1074/jbc.M301774200 . 12730199

[81] Carrano AC, Eytan E, Hershko A, Pagano M. SKP2 is required for ubiquitin-mediated degradation of the CDK inhibitor p27. Nat Cell Biol 1999;1(4):193–9. Epub 1999/11/13. 10.1038/12013 . 10559916

[82] Zhao H, Bauzon F, Fu H, Lu Z, Cui J, Nakayama K, et al. Skp2 deletion unmasks a p27 safeguard that blocks tumorigenesis in the absence of pRb and p53 tumor suppressors. Cancer cell. 2013;24(5):645–59. Epub 2013/11/16. 10.1016/j.ccr.2013.09.021 24229711 PMC3880806

[83] Miller TE, Liau BB, Wallace LC, Morton AR, Xie Q, Dixit D, et al. Transcription elongation factors represent in vivo cancer dependencies in glioblastoma. Nature. 2017;547(7663):355–9. Epub 2017/07/06. 10.1038/nature23000 28678782 PMC5896562

[84] Chow LM, Endersby R, Zhu X, Rankin S, Qu C, Zhang J, et al. Cooperativity within and among Pten, p53, and Rb pathways induces high-grade astrocytoma in adult brain. Cancer cell. 2011;19(3):305–16. Epub 2011/03/15. 10.1016/j.ccr.2011.01.039 21397855 PMC3060664

[85] Filtz EA, Emery A, Lu H, Forster CL, Karasch C, Hallstrom TC. Rb1 and Pten Co-Deletion in Osteoblast Precursor Cells Causes Rapid Lipoma Formation in Mice. PLoS One. 2015;10(8):e0136729. Epub 2015/09/01. 10.1371/journal.pone.0136729 26317218 PMC4552947

[86] Xie C, Lu H, Nomura A, Hanse EA, Forster CL, Parker JB, et al. Co-deleting Pten with Rb in retinal progenitor cells in mice results in fully penetrant bilateral retinoblastomas. Molecular cancer. 2015;14:93. Epub 2015/04/25. 10.1186/s12943-015-0360-y 25907958 PMC4411757

[87] Hill R, Song Y, Cardiff RD, Van Dyke T. Heterogeneous tumor evolution initiated by loss of pRb function in a preclinical prostate cancer model. Cancer Res 2005;65(22):10243–54. Epub 2005/11/17. 10.1158/0008-5472.CAN-05-1579 . 16288012

[88] Ku SY, Rosario S, Wang Y, Mu P, Seshadri M, Goodrich ZW, et al. Rb1 and Trp53 cooperate to suppress prostate cancer lineage plasticity, metastasis, and antiandrogen resistance. Science (New York, NY. 2017;355(6320):78–83. Epub 2017/01/07. 10.1126/science.aah4199 28059767 PMC5367887

[89] Koh J, Enders GH, Dynlacht BD, Harlow E. Tumour-derived p16 alleles encoding proteins defective in cell-cycle inhibition. Nature 1995;375(6531):506–10. Epub 1995/06/08. 10.1038/375506a0 . 7777061

[90] Monahan KB, Rozenberg GI, Krishnamurthy J, Johnson SM, Liu W, Bradford MK, et al. Somatic p16(INK4a) loss accelerates melanomagenesis. Oncogene. 2010;29(43):5809–17. Epub 2010/08/11. 10.1038/onc.2010.314 20697345 PMC3007178

[91] Sewastianik T, Jiang M, Sukhdeo K, Patel SS, Roberts K, Kang Y, et al. Constitutive Ras signaling and Ink4a/Arf inactivation cooperate during the development of B-ALL in mice. Blood Adv. 2017;1(25):2361–74. Epub 2018/01/04. 10.1182/bloodadvances.2017012211 29296886 PMC5729631

[92] Fisher GH, Wellen SL, Klimstra D, Lenczowski JM, Tichelaar JW, Lizak MJ, et al. Induction and apoptotic regression of lung adenocarcinomas by regulation of a K-Ras transgene in the presence and absence of tumor suppressor genes. Genes & development. 2001;15(24):3249–62. Epub 2001/12/26. 10.1101/gad.947701 11751631 PMC312852

[93] Hu Y, Sopko R, Foos M, Kelley C, Flockhart I, Ammeux N, et al. FlyPrimerBank: an online database for Drosophila melanogaster gene expression analysis and knockdown evaluation of RNAi reagents. G3 (Bethesda). 2013;3(9):1607–16. Epub 2013/07/31. 10.1534/g3.113.007021 23893746 PMC3755921

[94] Hu Y, Comjean A, Perkins LA, Perrimon N, Mohr SE. GLAD: an Online Database of Gene List Annotation for Drosophila. J Genomics. 2015;3:75–81. Epub 2015/07/15. 10.7150/jgen.12863 26157507 PMC4495321

[95] Franz M, Lopes CT, Huck G, Dong Y, Sumer O, Bader GD. Cytoscape.js: a graph theory library for visualisation and analysis. Bioinformatics. 2016;32(2):309–11. Epub 2015/09/30. 10.1093/bioinformatics/btv557 26415722 PMC4708103

[96] Hu Y, Flockhart I, Vinayagam A, Bergwitz C, Berger B, Perrimon N, et al. An integrative approach to ortholog prediction for disease-focused and other functional studies. BMC bioinformatics. 2011;12:357. Epub 2011/09/02. 10.1186/1471-2105-12-357 21880147 PMC3179972

[97] Vinayagam A, Hu Y, Kulkarni M, Roesel C, Sopko R, Mohr SE, et al. Protein complex-based analysis framework for high-throughput data sets. Science signaling. 2013;6(264):rs5. Epub 2013/02/28. 10.1126/scisignal.2003629 23443684 PMC3756668

[98] Lord CJ, Quinn N, Ryan CJ. Integrative analysis of large-scale loss-of-function screens identifies robust cancer-associated genetic interactions. eLife. 2020;9. Epub 2020/05/29. 10.7554/eLife.58925 32463358 PMC7289598

[99] Corsello SM, Nagari RT, Spangler RD, Rossen J, Kocak M, Bryan JG, et al. Discovering the anti-cancer potential of non-oncology drugs by systematic viability profiling. Nat Cancer. 2020;1(2):235–48. Epub 2020/07/03. 10.1038/s43018-019-0018-6 32613204 PMC7328899

[100] Ghandi M, Huang FW, Jane-Valbuena J, Kryukov GV, Lo CC, McDonald ER, 3rd, et al. Next-generation characterization of the Cancer Cell Line Encyclopedia. Nature. 2019;569(7757):503–8. Epub 2019/05/10. 10.1038/s41586-019-1186-3 31068700 PMC6697103

[101] Benjamini Y, Hochberg Y. Controlling the False Discovery Rate: A Practical and Powerful Approach to Multiple Testing. Journal of the Royal Statistical Society: Series B (Methodological). 1995;57 (1):289–300. 10.1111/j.2517-6161.1995.tb02031.x

