# Supplementary figures and images for: Cross-species identification of PIP5K1-, splicing- and ubiquitin-related pathways as potential targets for RB1-deficient cells
Source: PLoS Genet. 2021 Feb 16;17(2):e1009354. doi: 10.1371/journal.pgen.1009354 (PMC7909629; doi:10.1371/journal.pgen.1009354)

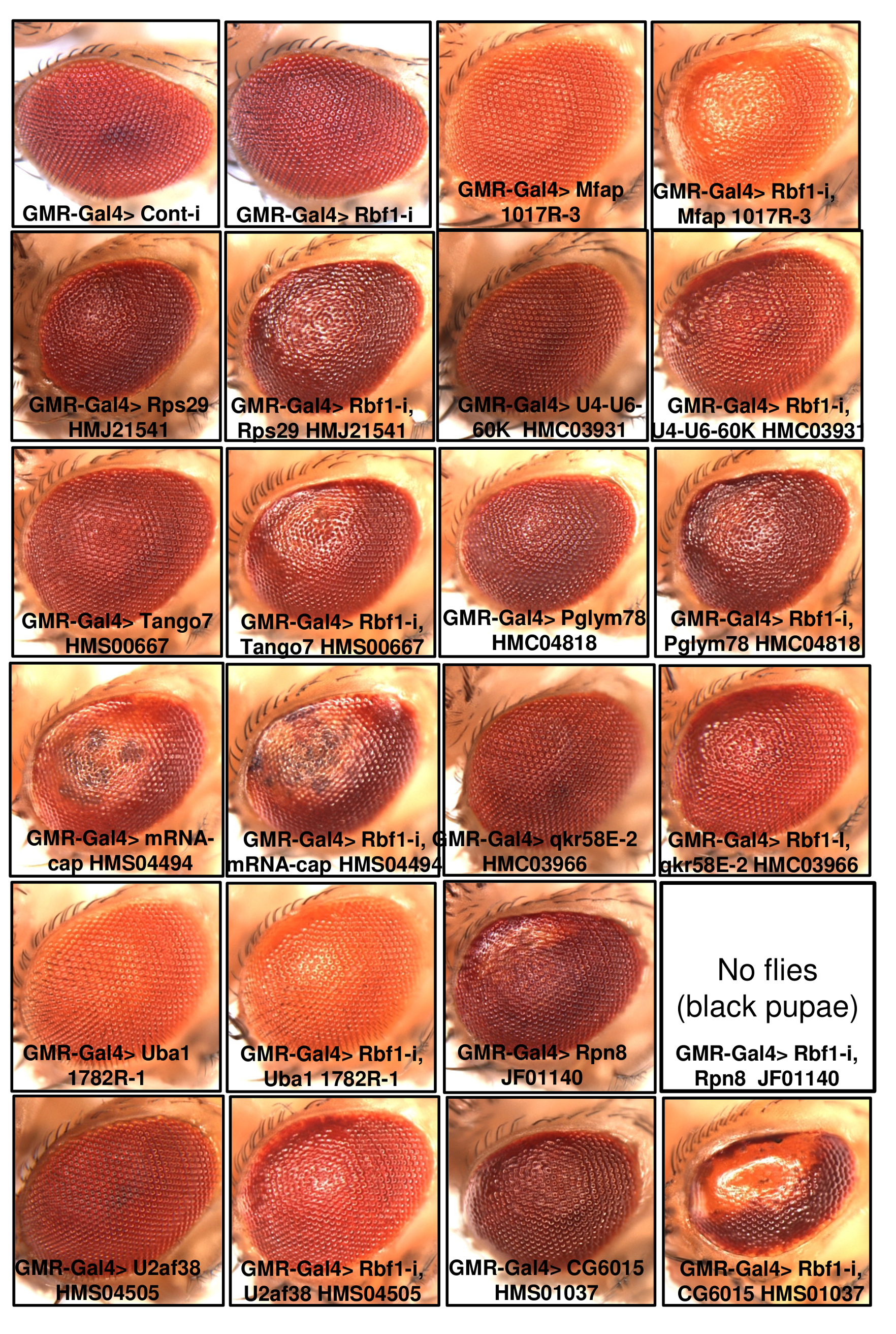

Supplement: S1 Fig — It should be noted that the first two control pictures GMR-Gal4> Contr RNAi and GMR-Gal4> Rbf1-i are similar at all supplemental figures and similar to Fig 1B and 1C. They are added for easier comparison of phenotypes between different figures. (TIF) [file pgen.1009354.s001.tif]

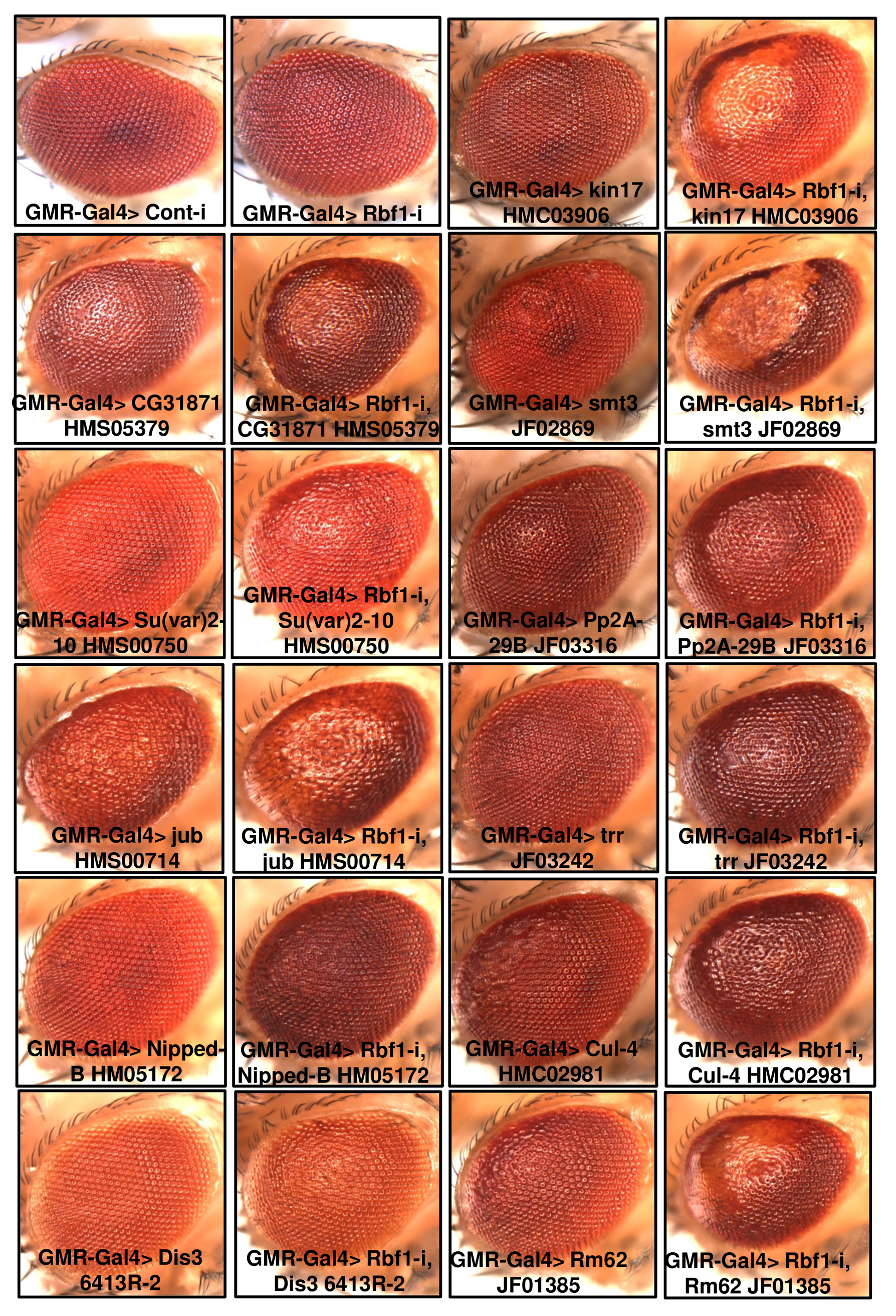

Supplement: S2 Fig — It should be noted that the first two control pictures GMR-Gal4> Contr RNAi and GMR-Gal4> Rbf1-i are similar at all supplemental figures and similar to Fig 1B and 1C. They are added for easier comparison of phenotypes between different figures. (TIF) [file pgen.1009354.s002.tif]

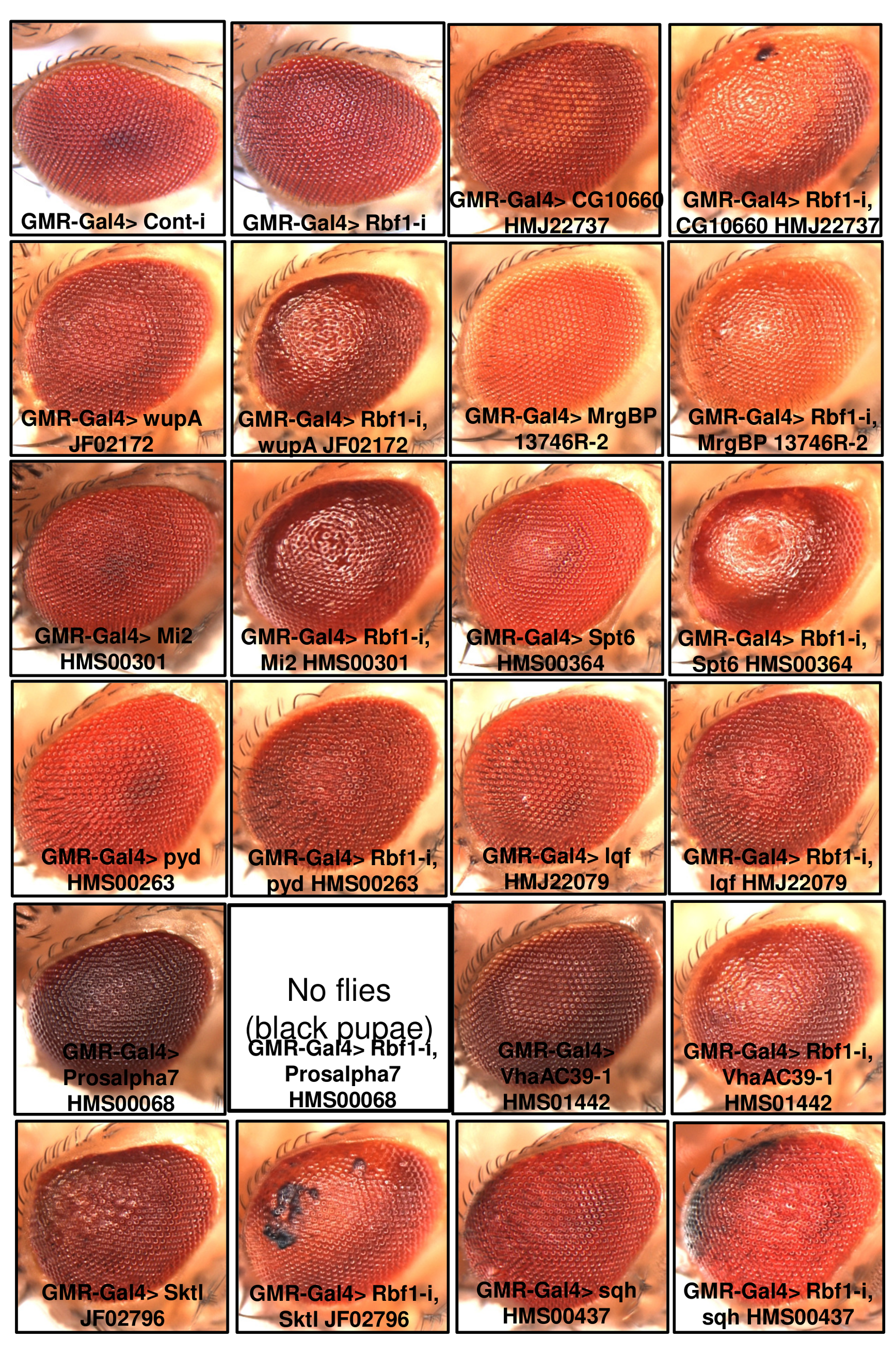

Supplement: S3 Fig — It should be noted that the first two control pictures GMR-Gal4> Contr RNAi and GMR-Gal4> Rbf1-i are similar at all supplemental figures and similar to Fig 1B and 1C. They are added for easier comparison of phenotypes between different figures. (TIF) [file pgen.1009354.s003.tif]

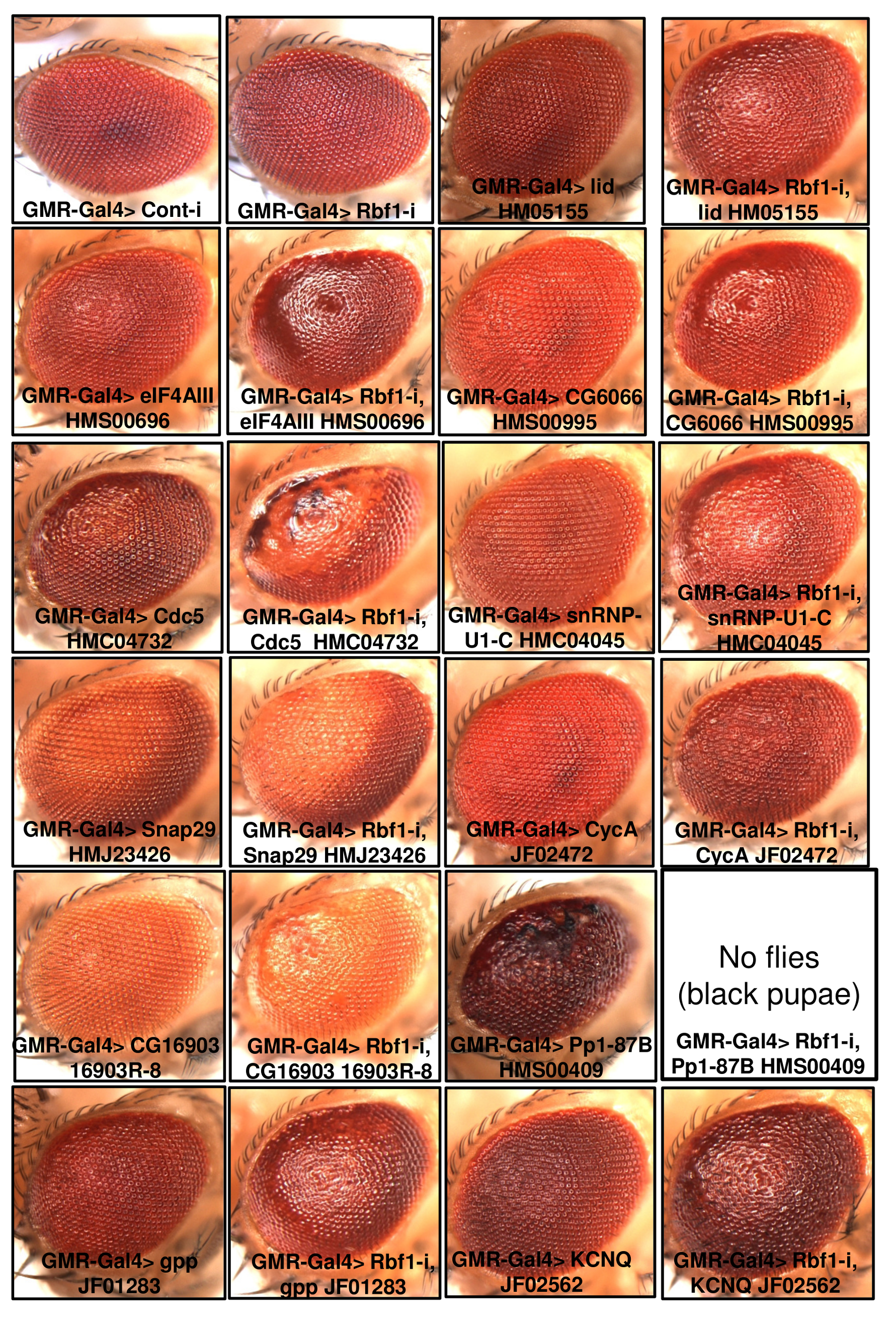

Supplement: S4 Fig — It should be noted that the first two control pictures GMR-Gal4> Contr RNAi and GMR-Gal4> Rbf1-i are similar at all supplemental figures and similar to Fig 1B and 1C. They are added for easier comparison of phenotypes between different figures. (TIF) [file pgen.1009354.s004.tif]

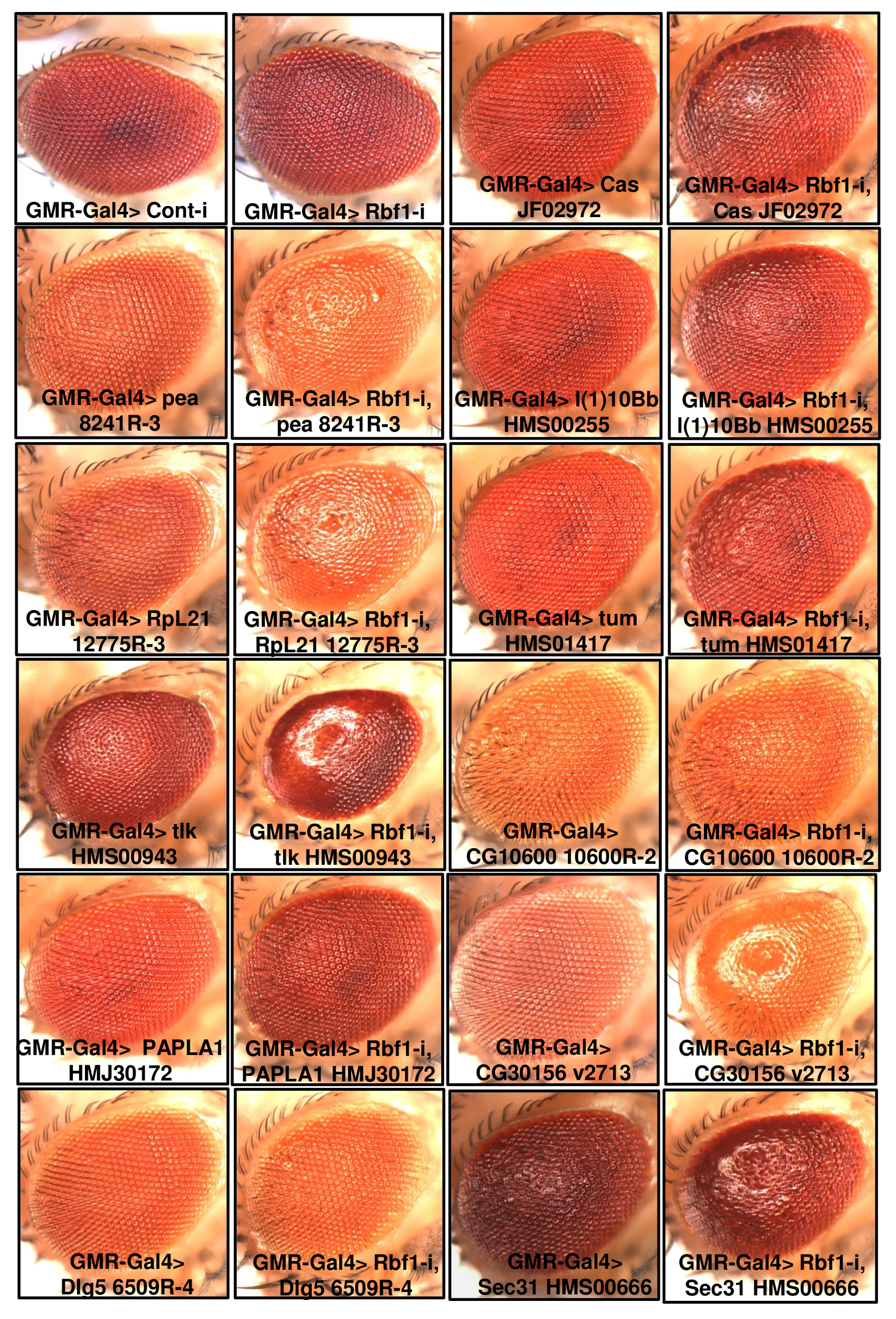

Supplement: S5 Fig — It should be noted that the first two control pictures GMR-Gal4> Contr RNAi and GMR-Gal4> Rbf1-i are similar at all supplemental figures and similar to Fig 1B and 1C. They are added for easier comparison of phenotypes between different figures. (TIF) [file pgen.1009354.s005.tif]

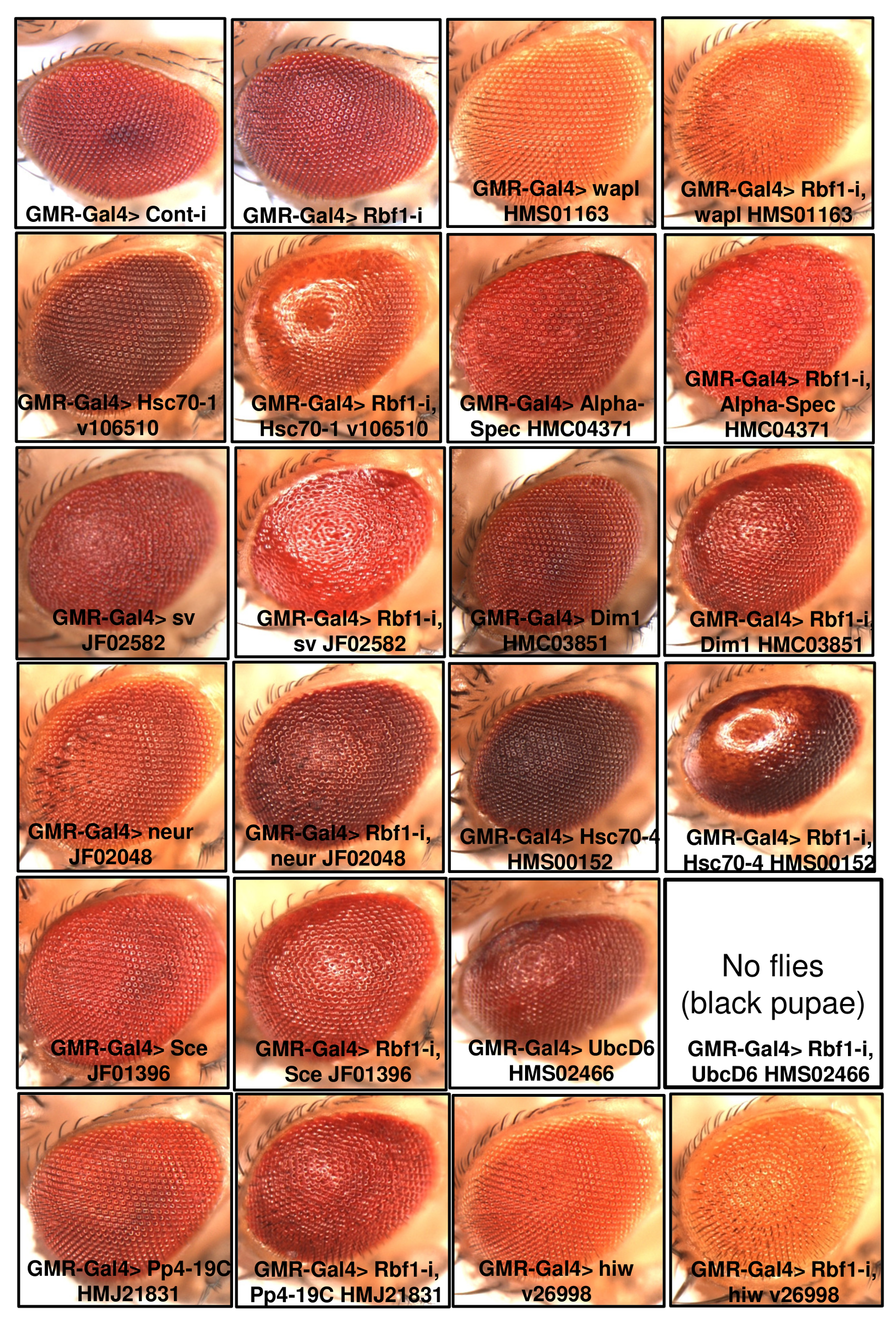

Supplement: S6 Fig — It should be noted that the first two control pictures GMR-Gal4> Contr RNAi and GMR-Gal4> Rbf1-i are similar at all supplemental figures and similar to Fig 1B and 1C. They are added for easier comparison of phenotypes between different figures. (TIF) [file pgen.1009354.s006.tif]

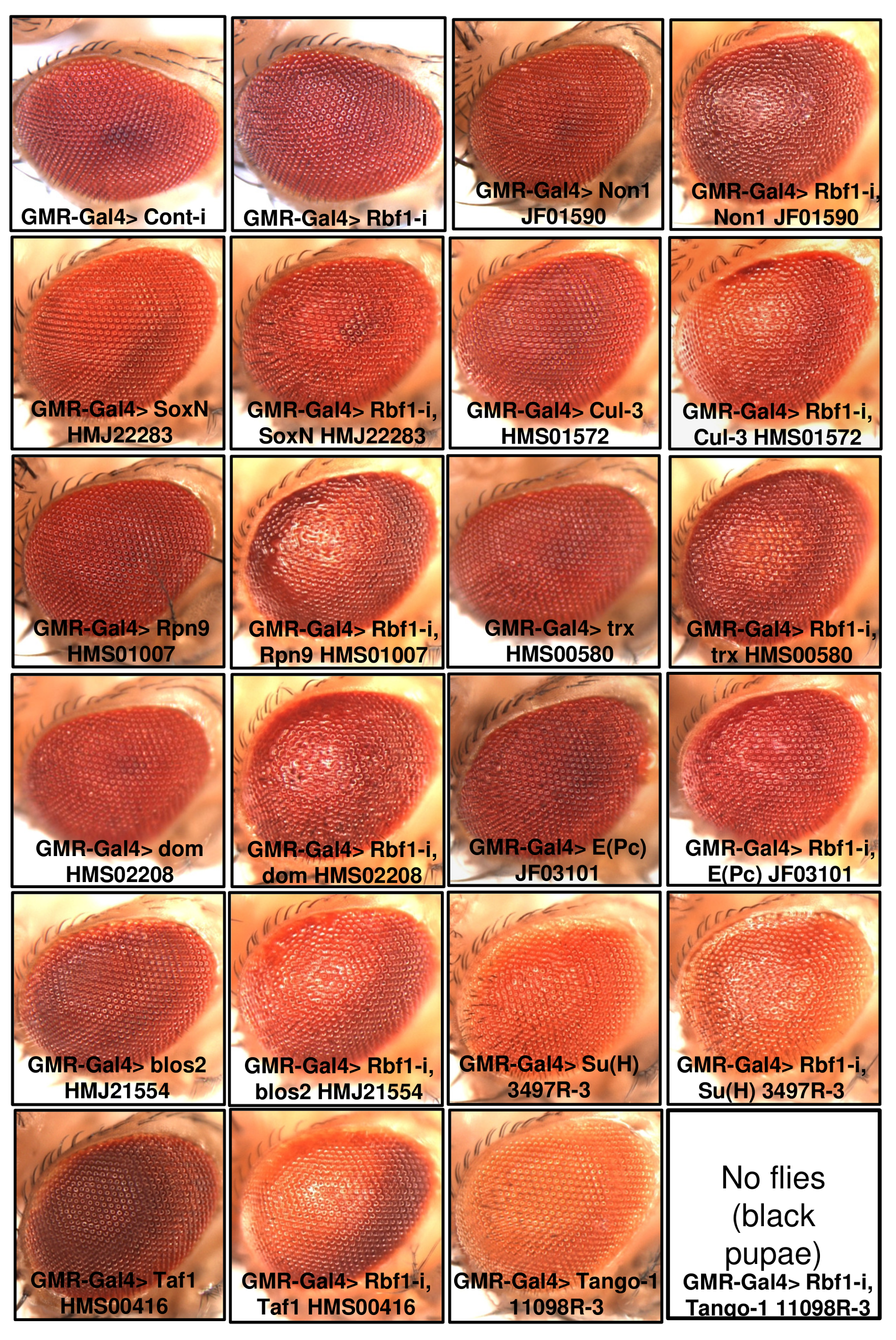

Supplement: S7 Fig — It should be noted that the first two control pictures GMR-Gal4> Contr RNAi and GMR-Gal4> Rbf1-i are similar at all supplemental figures and similar to Fig 1B and 1C. They are added for easier comparison of phenotypes between different figures. (TIF) [file pgen.1009354.s007.tif]

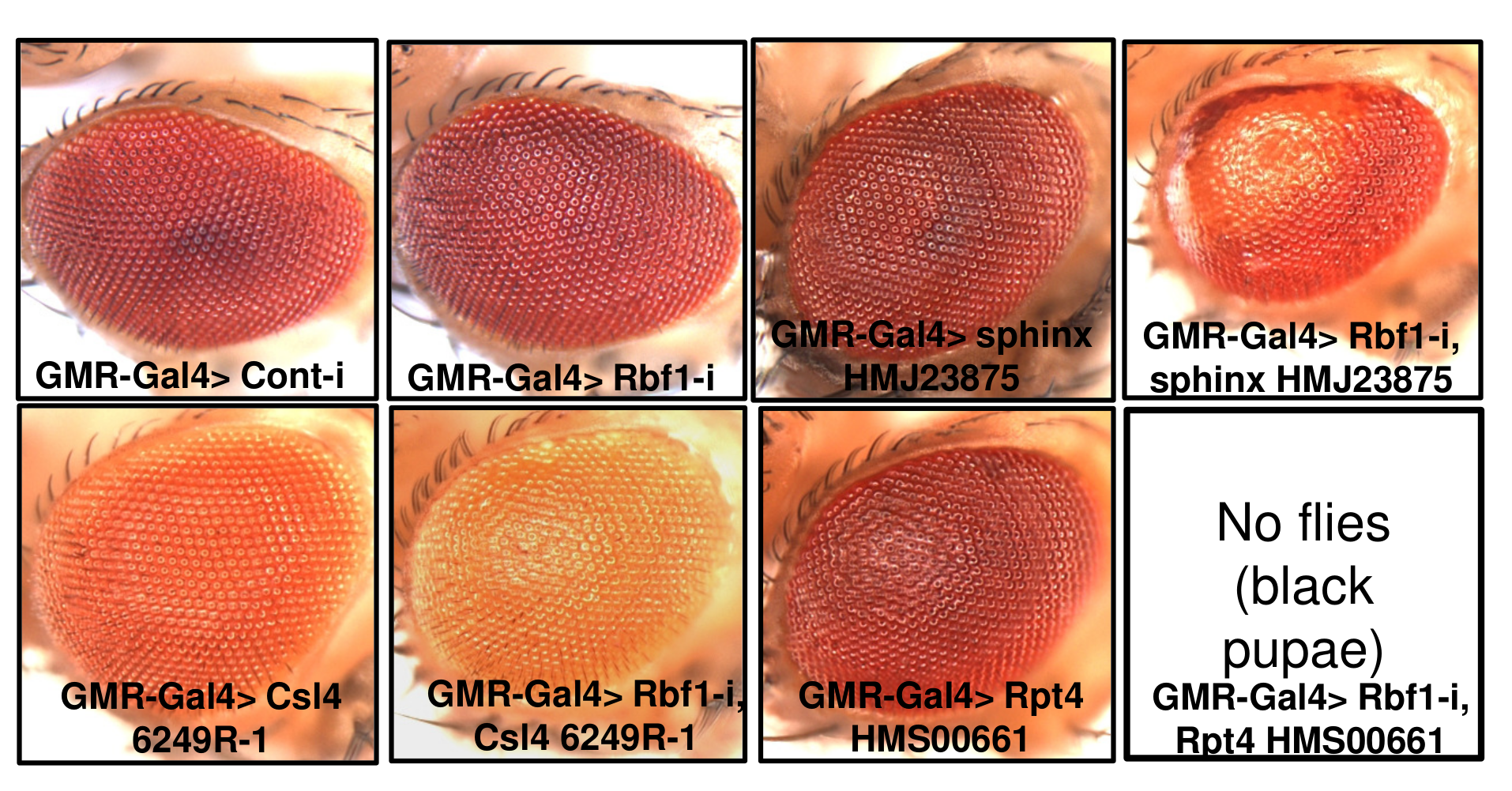

Supplement: S8 Fig — It should be noted that the first two control pictures GMR-Gal4> Contr RNAi and GMR-Gal4> Rbf1-i are similar at all supplemental figures and similar to Fig 1B and 1C. They are added for easier comparison of phenotypes between different figures. (TIF) [file pgen.1009354.s008.tif]

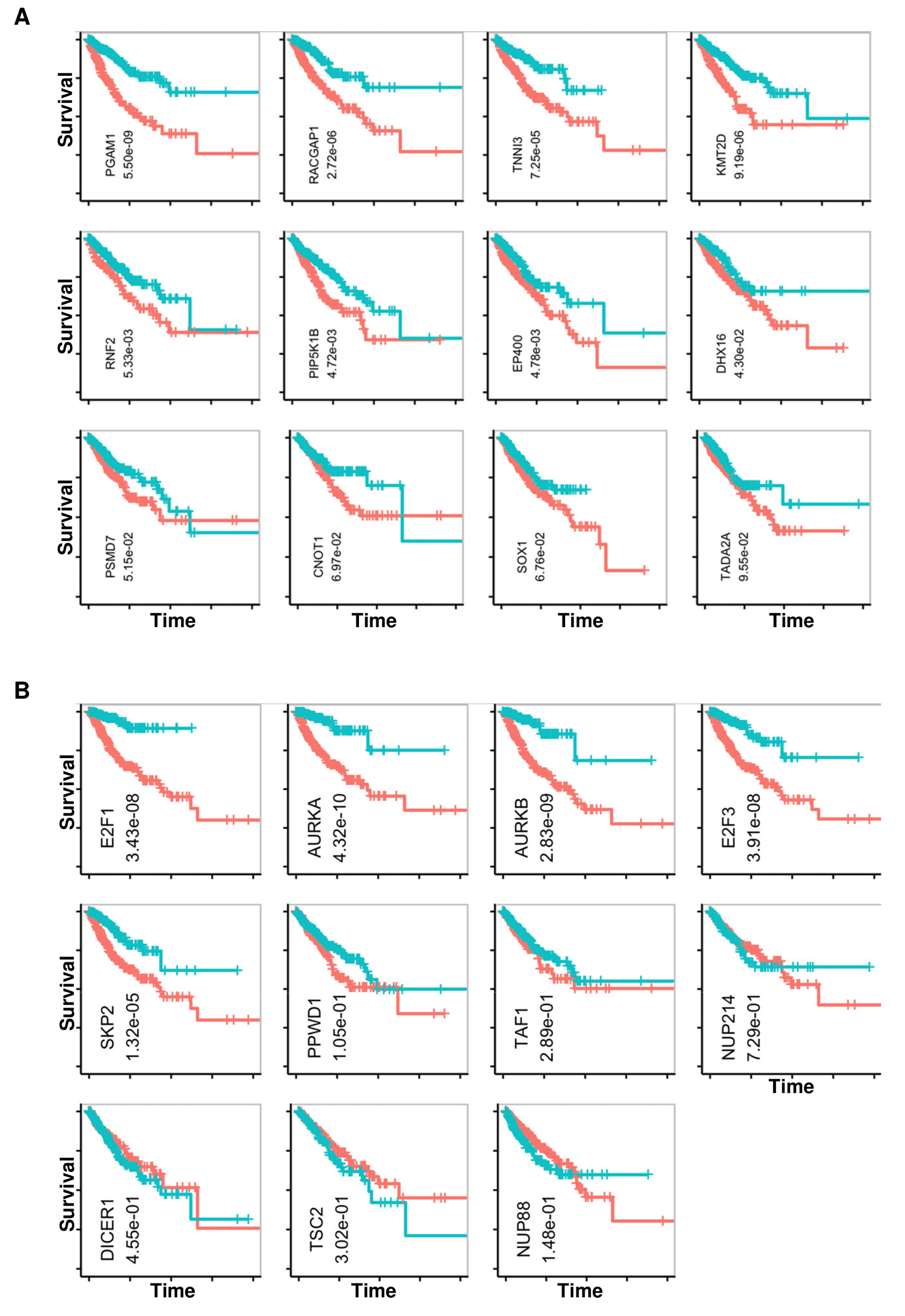

Supplement: S9 Fig — Kaplan Meier’s survival curves along with corrected p-values for 12 genes that showed SL with RB1 in cancer patients and in one of human cancer cell lines screens (A). Kaplan Meier’s survival curves along with corrected p-values for 11 genes positive controls from literature (B). Blue line–levels of both genes (RB1 and outlined gene) are low, red line–only RB1 level is low. (TIF) [file pgen.1009354.s009.tif]

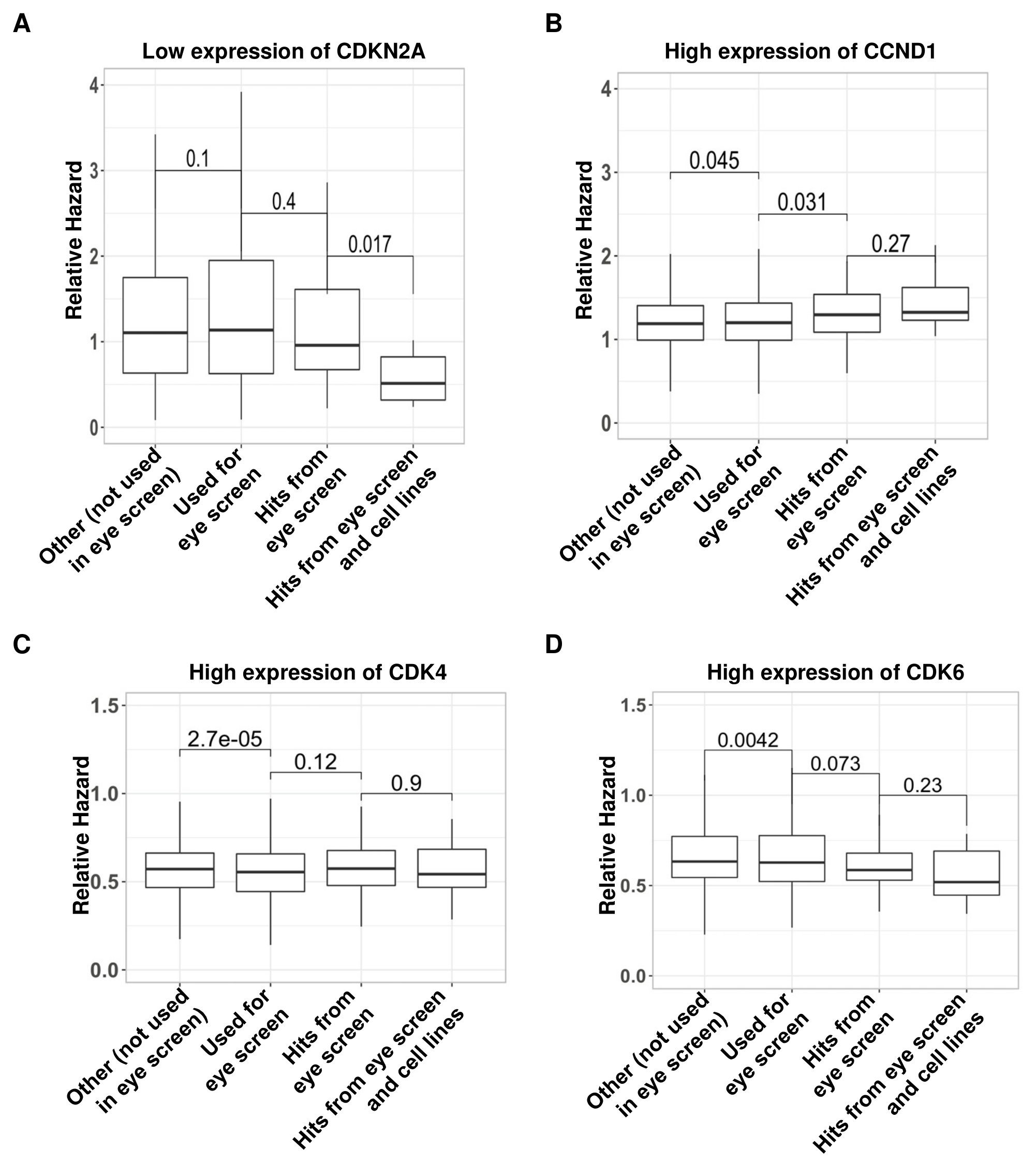

Supplement: S10 Fig — The overall survival of the TCGA cancer patients by comparing the patients with high activity of CCND1 (B), CDK4 (C), or CDK6 (D) and low activity of genes from the screen against the high activity of only CCND1 (B), CDK4 (C), or CDK6 (D) in lung, breast, and prostate cancers patients. Box plot comparing the relative hazard computed using log-rank test for different gene sets. (TIF) [file pgen.1009354.s010.tif]

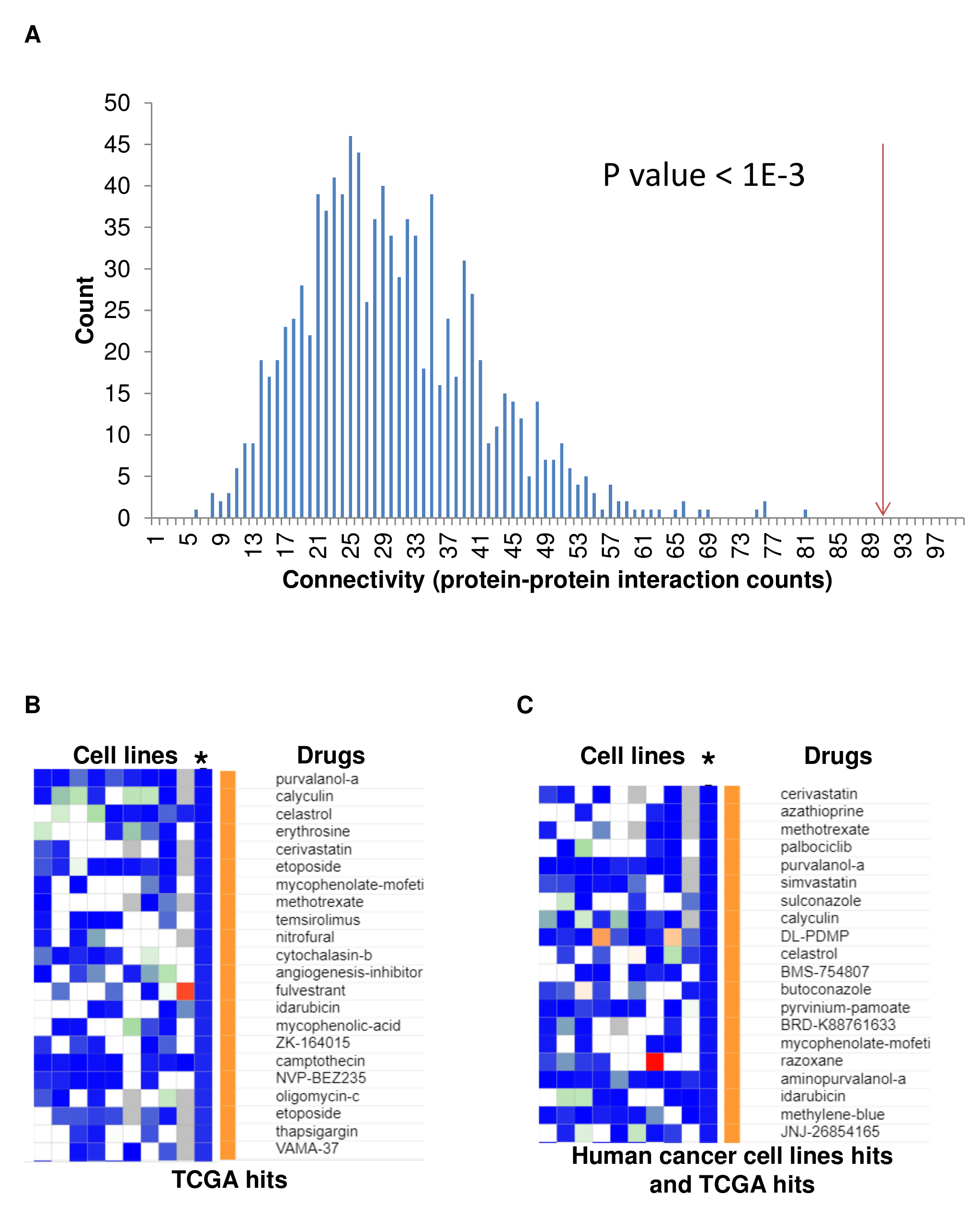

Supplement: S11 Fig — The distribution of connectivity for 1000 lists of randomly selected 95 genes from all the genes screened (A). Top 20 drugs from the connectivity map analysis that scored from the analysis of subnetwork 2 that contains proteins that also scored as SL partners in TCGA human cancer patients (B). Top 20 drugs from the connectivity map analysis of proteins that scored as SL partners in both human cancer cell lines and TCGA human cancer patients (C). (TIF) [file pgen.1009354.s011.tif]

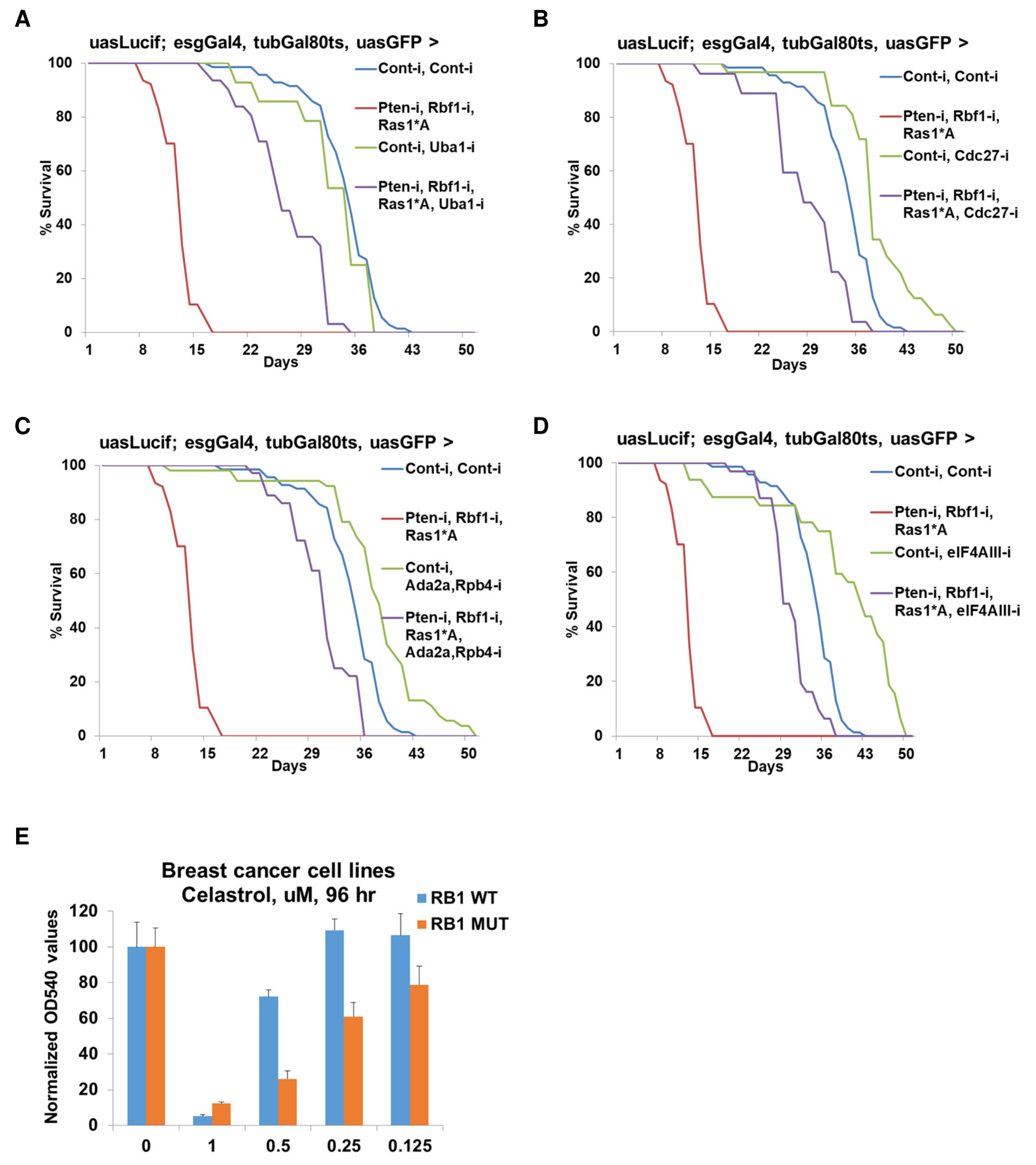

Supplement: S12 Fig — Lifespan analysis of UAS-Luciferase, esg-Gal4, tubulinGal80ts, UAS-GFP lines containing either control RNAi (control) or combination of Rbf1 RNAi, Pten RNAi, Ras1A overexpression and crossed to either control RNAi or RNAi against Uba1 (A), Cdc27 (B), Ada2a/Rpb4 (C), or eIF4AIII (D). Note that the lifespans of control RNAi flies are the same between different panels. Proliferation of RB1 wild-type and RB1 mutant breast cancer cells treated with 1, 0.5, 0.25, or 0.125 uM of celastrol for 96 hr (crystal violet staining). Data are shown as means ± SD. (E). (TIF) [file pgen.1009354.s012.tif]
